# New extended distribution-free homogenously weighted monitoring schemes for monitoring abrupt shifts in the location parameter

**DOI:** 10.1371/journal.pone.0261217

**Published:** 2022-01-21

**Authors:** Tokelo Irene Letshedi, Jean-Claude Malela-Majika, Sandile Charles Shongwe

**Affiliations:** 1 Department of Statistics, College of Science, Engineering and Technology, University of South Africa, Pretoria, South Africa; 2 Department of Statistics, Faculty of Natural and Agricultural Sciences, University of Pretoria, Hatfield, South Africa; 3 Department of Mathematical Statistics and Actuarial Science, Faculty of Natural and Agricultural Sciences, University of the Free State, Bloemfontein, South Africa; Wright State University, UNITED STATES

## Abstract

A homogeneously weighted moving average (HWMA) monitoring scheme is a recently proposed memory-type scheme that gained its popularity because of its simplicity and superiority over the exponentially weighted moving average (EWMA) and cumulative sum (CUSUM) schemes in detecting small disturbances in the process. Most of the existing HWMA schemes are designed based on the assumption of normality. It is well-known that the performance of such monitoring schemes degrades significantly when this assumption is violated. Therefore, in this paper, three distribution-free monitoring schemes are developed based on the Wilcoxon rank-sum *W* statistic. First, the HWMA *W* scheme is introduced. Secondly, the double HWMA (DHWMA) *W* scheme is proposed to improve the ability of the HWMA *W* scheme in detecting very small disturbances in the location parameter and at last, the hybrid HWMA (HHWMA) *W* scheme is also proposed because of its flexibility and better performance in detecting shifts of different sizes. The zero-state performances of the proposed schemes are investigated using the characteristics of the run-length distribution. The proposed schemes outperform their existing competitors, i.e. EWMA, CUSUM and DEWMA *W* schemes, in many situations, and particularly the HHWMA *W* scheme is superior to these competitors regardless of the size of the shift in the location parameter. Real-life data are used to illustrate the implementation and application of the new monitoring schemes.

## 1. Introduction

There are numerous monitoring schemes documented in the statistical process monitoring (SPM) literature. Over the years, a fact has been proven that SPM tools are crucial tools to monitor most industrial processes to enhance the quality of products and services. Monitoring schemes are intended to monitor and identify unnatural (i.e., assignable) causes of variation as soon as they occur. In-control (IC) processes are favourable as they consist of common (or natural) causes of variations that cannot disturb the process to the extent of ruining the outputs. Walter A. Shewhart was the first to design a modern monitoring scheme in the 1920s, see the introductory chapter of [1]. Shewhart schemes are widely known to be responsive to large shifts in a process. However, they are relatively slow in detecting small to moderate shifts in a process. When it comes to a quick detection of small to moderate shifts, the SPM literature recommends the use of memory-type monitoring schemes such as the exponentially weighted moving average (EWMA) by [2] and the cumulative sum (CUSUM) by [3]. Memory-type schemes have their disadvantages of being slow in detecting large shifts. More contributions and modifications of the memory-type schemes have been recorded over the years (see, [4–13]; just to cite a few). More recently, [14] introduced a new memory-type scheme called the homogeneously weighted moving average (HWMA). The charting statistic of the HWMA scheme assigns a weight *λ* (i.e., a smoothing parameter, where 0 < *λ* < 1) to the current sample and a weight (1 − *λ*) is homogeneously (or equally) distributed to all the previous samples. Some recent publications on HWMA schemes can be found in [15–21].

Researchers have also introduced different combinations of control charts for better results in improving the quality of products and services for end users. The double EWMA (DEWMA) scheme was introduced by [4] to increase the sensitivity of the EWMA scheme in monitoring small to moderate shifts; see also, [22, 23]. Furthermore, the hybrid EWMA (HEWMA) scheme was first introduced by [24, 25] to also increase the sensitivity of EWMA scheme and it was shown that the DEWMA scheme is a special case of the HEWMA scheme. Similarly, the double HWMA (DHWMA) and hybrid HWMA (HHWMA) schemes were discussed in [26–29].

Firstly, it is important to mention that a monitoring scheme is defined as distribution-free if the IC run-length distribution is the same for every continuous distribution. Next, the abovementioned HWMA-type monitoring schemes are based on normally distributed data. However, there are some distribution-free HWMA-type schemes that have been discussed in the SPM literature. That is, [30] proposed the HWMA schemes based on the sign and signed rank statistics and more recently, [31] and [32] provided different designs of the DHWMA scheme based on the sign statistic. The sign and signed-rank monitoring schemes are applicable in situations where the true process location value is known. Note though, in most practical situations, the true process location value may not be known; which means that the use of simple sign and signed-rank statistics are invalid; hence, more complex nonparametric methods are applicable. These can be nonparametric methods based on the precedence, exceedance, Wilcoxon rank-sum, Mann-Whitney, etc.; see [33] for more details. The focus of this paper is on the Wilcoxon-rank-sum (*W*) test by [34]. In essence, the *W* test statistic compares the outcomes between two independently and identically distributed (i.i.d.) samples (or groups) and tests whether the two samples are likely derived from the same population distribution, i.e., the two samples are collected from a population with the same shape. For some discussions on monitoring schemes based on the *W* statistic, see [5, 35–40]. The limitations of the existing research works that motivated this paper are that the SPM literature publications are mostly populated with parametric monitoring schemes. However, in most real-life applications, data are not always normally distributed. Therefore, there is a need of robust monitoring schemes that do not rely on the parametric settings or assumption. Moreover, there is a need of more efficient memory-type monitoring schemes that will perform better regardless of the size of the shift and/or the smoothing parameter.

Note that there is a limited number of HWMA-type monitoring schemes in the literature. Table 1 present the summary and gaps of the HWMA-type schemes in the SPM literature. As it can be noticed, there are only two dozen of articles related to the HWMA scheme. These include parametric and nonparametric variable and attribute HWMA-related schemes. For attribute schemes, there is only one article by [41] and only four nonparametric HWMA schemes based on the sign, signed-rank and Lepage statistics (see [31, 32] and [42]). Thus, there is an increasing need in the design of attribute and parametric monitoring schemes to cater for processes that are based on discrete and non-normal distribution, respectively. For more recent articles on HWMA scheme, readers are referred to the articles by [41–48]. A summary and more details of the existing HWMA-related articles are presented in Table 1.

**Table 1:** Summary of the literature on HWMA-related monitoring schemes.

	Type of scheme		Type of scheme per Assumption		Quality characteristic			Parameter(s)		Type of Data		Process Type		Type of HWMA scheme
Paper	Variable	Attribute	Parametric	Nonparametric	Location	Variability	Profiles	Known	Unknown	Univariate	Multivariate	I.I.D.	Serial Dependence	
Abbas (2018)	✓		✓		✓			✓	✓	✓		✓		HWMA $\bar{X}$
Abbasi et al. (2021)	✓		✓			✓		✓		✓		✓		HWMA *S*
Adegoke et al. (2019a)	✓		✓		✓			✓	✓	✓			✓	HWMA $\bar{X}$ with AIB
Adegoke et al. (2019b)	✓		✓		✓			✓			✓	✓		HWMA *T*^2^
Nawaz and Han (2020)	✓		✓		✓			✓		✓		✓		HWMA $\bar{X}$ using RSS
Abbas et al. (2020)	✓		✓		✓				✓		✓	✓		HWMA *T*^2^
Abid et al. (2020a)	✓		✓		✓			✓	✓	✓		✓		DHWMA $\bar{X}$
Abid et al. (2020b)	✓		✓		✓			✓		✓		✓		HWMA-CUSUM $\bar{X}$
Adeoti and Koleoso (2020)	✓		✓		✓			✓		✓		✓		HHWMA $\bar{X}$
Adeoti et al. (2021)		✓	✓		✓	✓		✓		✓		✓		CMP-HWMA
Alevizakos et al. (2021)	✓		✓		✓			✓		✓		✓		DHWMA $\bar{X}$
Alevizakos et al. (2021)	✓			✓	✓			✓		✓		✓		DHWMA SN
Chan et al. (2021)	✓			✓		✓		✓		✓		✓		DEWMA and HWMA LP
Knoth et al. (2021)	✓		✓							✓		✓		HWMA and PM-type
Malela-Majika et al. (2021)	✓		✓		✓			✓		✓		✓		HHWMA $\bar{X}$
Raza et al. (2020)	✓			✓	✓			✓		✓		✓		HWMA SN & SR
Riaz et al. (2021)	✓			✓	✓			✓		✓		✓		DHWMA SN
Riaz et al. (2020)	✓		✓			✓		✓		✓		✓		HWMA *S*^2^
Dawod et al. (2020)	✓						✓		✓		✓		✓	HWMA profiles
Thanwane et al. (2021a)	✓		✓		✓			✓		✓		✓		HWMA $\bar{X}$ with ME
Thanwane et al. (2020)	✓		✓		✓				✓	✓			✓	HWMA $\bar{X}$ with AC&ME
Thanwane et al. (2021b)	✓		✓		✓				✓	✓		✓		HWMA $\bar{X}$ with FIR
Thanwane et al. (2021c)	✓		✓		✓			✓		✓		✓	✓	HWMA with AC&ME
Thanwane et al. (2021d)	✓		✓		✓				✓	✓		✓		HWMA $\bar{X}$ with ME

Note: I.I.D. = independent and identically distributed; CMP = Conway-Maxwell Poisson; LP = Lepage statistic; PM = Progressive Mean; AC = Autocorrelation; ME = Measurement Errors; AIB = Auxiliary Information-Based; FIR = Fast Initial Response; SN = Sign; SR = Signed-Rank; RSS = Rank Set Sampling.

Therefore, the objective of this paper is to propose *three* new distribution-free HWMA-type monitoring schemes based on the two-sample *W* statistic. These are the basic HWMA *W* scheme, DHWMA *W* scheme and the HHWMA *W* scheme. Moreover, the run-length properties of the latter schemes are derived and evaluated using Monte Carlo simulations. The run-length performance of the HWMA, DHWMA and HHWMA *W* schemes are compared to each other and thereafter, the corresponding existing competitors (i.e., EWMA and CUSUM *W* schemes by [5]; DEWMA *W* scheme by [49]) are also compared to the newly proposed monitoring schemes. Thus, the contribution of this paper can be summarised as follows:

- The design of robust and efficient monitoring schemes under the violation of the normality assumption.
- The introduction of new HWMA, DHWMA and HHWMA schemes based on the WRS statistic.
- The investigation of both specific and overall performances of the proposed HWMA-type monitoring scheme since most of the existing ones are investigated using specific measures of performance.

The rest of this paper is organised as follows: Section 2 introduces the *W* statistic and the fundamental concepts of the proposed HWMA, DHWMA and HHWMA *W* schemes. The operation and implementation steps of the proposed schemes are presented in Section 3. Section 4 presents the performance analysis of the proposed schemes. Moreover, the HWMA, DHWMA and HHWMA *W* schemes are further compared to the existing CUSUM, EWMA and DEWMA *W* schemes. An illustrative example using real-life data is provided in Section 5, and finally, Section 6 presents the concluding remarks, recommendations and future research works.

## 2. The proposed HWMA *W* monitoring scheme and its extended versions

### 2.1 The Wilcoxon rank sum *W* statistic

Assuming that *X* = {*x*_*i*_: *i* = 1,2, …, *m*} is IC reference sample (i.e., phase I) of size *m* and *Y* = {*y*_*tj*_: *t* = 1,2, …; *j* = 1,2,…,*n*} a test sample (i.e., phase II) of size *n*. The samples *X* and *Y* are independent from one another and from the samples’ observations with unknown cumulative distribution function (c.d.f.), denoted as *F*(*x*) and *G*(*y*), respectively. The test and reference samples use the same distribution, but they differ in the location shift (*δ*). Thus, when *δ* = 0, the two distributions are the same (i.e., *F* = *G*) and the process is considered to be IC. Otherwise, the process is considered to be out-of-control (OOC).

The *W* statistic was proposed by [34] to compare two independent samples in order to check whether their populations mean ranks differ. The *W* statistic for a phase I sample of size *m* and a phase II sample of size *n* is given by

$$W_{SRS_{t}}=\overset{N}{\underset{z=1}{\sum }}z\cdot \left(I_{z}\right),\;t=1,\;2,\;3,\ldots ,$$ (1)

where the indicator *I*_*Z*_ = 1 when the *z*^*th*^ observation of the ordered *N* (with *N* = *m* + *n*) observations obtained after combining the phase I and phase II samples is from the phase I sample and *I*_*Z*_ = 0 when the *z*^*th*^ observation is from the phase II sample.

Assuming that no ties are observed, then the mean and the variance of the *W* statistic are given by (see [5])

$$\begin{array}{l} \mu_{W}=\frac{n\;\left(m+n+1\right)}{2}\\ \text{and}\\ \sigma_{W}^{2}=\frac{mn\left(m+n+1\right)}{12}, \end{array}$$ (2)

respectively.

### 2.2 The HWMA *W* scheme

The proposed HWMA *W* monitoring scheme is designed following [14]’s idea. Thus, the HWMA *W* statistic is defined by

$$H_{t}=\lambda {\text{W}}_{t}+\left(1-\lambda \right){\bar{W}}_{t-1},$$ (3)

where *λ* ∈ (0,1] is the smoothing parameter and ${\bar{W}}_{t-1}$ is the mean of the lagged *W*_*t*_ statistics (i.e., ${\bar{W}}_{t-1}=\sum_{S=1}^{t-1}{\text{W}}_{s}/\left(t-1\right)$) and ${\bar{W}}_{0}$ is set to be equal to *μ*_*W*_.

Thus, the mean and variance of *H*_*t*_ statistic are defined as follows:

$$E\left(H_{t}\right)=\mu_{H_{t}}=\mu_{W}$$ (4)

and

$$Var\left(H_{t}\right)=\{\begin{array}{c} \lambda^{2}\sigma_{W}^{2}\;for\;t=1\\ \left[\lambda^{2}+\frac{{\left(1-\lambda \right)}^{2}}{t-1}\right]\sigma_{W}^{2},\;for\;t>1 \end{array}$$ (5)

where $\sigma_{W}^{2}$ is defined in Eq (2). For more details on the derivation of the mean and variance of the *H*_*t*_ statistic, readers are referred to S1 Appendix of this paper. Using the above properties, the time-varying upper and lower control limits (*UCL* and *LCL*) of the HWMA *W* scheme are then given as

$$UCL_{t}/LCL_{t}=\{\begin{array}{c} \mu_{W}\pm L_{H}\;\lambda \sigma_{W}\;for\;t=1\\ \mu_{W}\pm L_{H}\sigma_{W}\sqrt{\left[\lambda^{2}+\frac{{\left(1-\lambda \right)}^{2}}{t-1}\right]},\;for\;t>1 \end{array}$$ (6)

where *L*_*H*_(*L*_*H*_ > 0) is the control limit constant of the HWMA *W* scheme which is determined such that the attained IC average run-length (*ARL*) is much closer or equal to the prespecified nominal IC *ARL* (*ARL*_0_) value. The center line is equal to *μ*_*W*_ (i.e., *CL* = *μ*_*W*_). Hence, the HWMA *W* scheme gives an OOC signal if the *H*_*t*_ statistic plots beyond the control limits defined in Eq (6).

### 2.3 The DHWMA *W* scheme

The DHWMA scheme for monitoring the process mean was proposed by [28]. In this paper, the DHWMA scheme is designed using the *W* statistic, and it is denoted as DHWMA *W* scheme. The charting statistic of the DHWMA *W* statistic is defined by

$$DH_{t}=\lambda H_{t}+(1-\lambda ){\bar{H}}_{t-1},$$ (7)

where *H*_*t*_ is defined in Eq (3), ${\overset{-}{H}}_{0}=\mu_{W}$ and ${\overset{-}{H}}_{t-1}$ is defined as

$${\bar{H}}_{t-1}=\frac{\sum_{s=1}^{t-1}H_{s}}{t-1}.$$ (8)

Thus, at a sampling time *t*, the mean and variance of the *DH*_*t*_ statistic are given by

$$E\left(DH_{t}\right)=\mu_{W}$$ (9)

and

$$Var\left(DH_{t}\right)=\left\{ \begin{array}{l} \lambda^{4}\sigma_{W}^{2}\;for\;t=1\\ \lambda^{2}\left(\lambda^{2}+4{\left(1-\lambda \right)}^{2}\right)\;\sigma_{W}^{2}\;for\;t=2\\ {}[\lambda^{4}+\frac{4\lambda^{2}{\left(1-\lambda \right)}^{2}}{{\left(t-1\right)}^{2}}+\frac{{\left(1-\lambda \right)}^{2}}{{\left(t-1\right)}^{2}}\overset{t-2}{\underset{u=1}{\sum }}{\left(2\lambda +\left(1-\lambda \right)\overset{t-2}{\underset{k=u}{\sum }}\frac{1}{k}\right)}^{2}]\sigma_{W}^{2},\;for\;t>2 \end{array}\right.$$ (10)

respectively. The expressions of the mean and variance of the DHWMA *W* statistic are derived in S2 Appendix.

Thus, the control limits of the DHWMA *W* scheme are defined by

$$DUCL_{t}/DLCL_{t}=\mu_{W}\pm L_{DH}\sqrt{Var(DH_{t})}$$ (11)

where *L*_*DH*_ (*L*_*DH*_ > 0) is the is the control limit constant of the DHWMA *W* scheme. The center line is equal to *μ*_*W*_ (i.e., *CL* = *μ*_*W*_). Hence, the DHWMA *W* scheme gives an OOC signal if the *DH*_*t*_ statistic plots beyond the control limits defined in Eq (11).

### 2.4 The HHWMA *W* scheme

This paper also proposes the HHWMA monitoring scheme using the *W* statistic and it is denoted as HHWMA *W* scheme. The charting statistic of the HHWMA *W* statistic is defined by

$$HH_{t}=\lambda_{1}H_{t}+\left(1-\lambda_{1}\right){\bar{H}}_{t-1},$$ (12)

where $H_{t}=\lambda_{2}{\text{W}}_{t}+\left(1-\lambda_{2}\right){\bar{W}}_{t-1}$ and ${\bar{H}}_{t-1}$ is defined in Eq (8) and ${\bar{H}}_{0}=$ *μ*_*W*_ as explained earlier. Note that this scheme has two different smoothing parameters, i.e., λ_1_ and λ_2_, which are defined in the interval (0,1]. When λ_1_ = λ_2_, then the HHWMA *W* scheme reduces to the DHWMA *W* scheme.

Thus, at sampling time *t*, the mean and variance of *HH*_*t*_ statistic are defined by

$$E\left(HH_{t}\right)=\mu_{W}$$ (13)

and

$$Var\left(HH_{t}\right)=\left\{ \begin{array}{l} \lambda_{1}^{2}\lambda_{2}^{2}\sigma_{W}^{2}\;\text{for}\;t=1\\ \left(\lambda_{1}^{2}\lambda_{2}^{2}+{\left(\lambda_{1}+\lambda_{2}-2\lambda_{1}\lambda_{2}\right)}^{2}\right)\sigma_{W}^{2}\;\text{for}\;t=2\\ {}[\lambda_{1}^{2}\lambda_{2}^{2}+\frac{{\left(\lambda_{1}+\lambda_{2}-2\lambda_{1}\lambda_{2}\right)}^{2}}{{\left(t-1\right)}^{2}}+\frac{1}{{\left(t-1\right)}^{2}}\sum_{u=1}^{t-2}{\left(\lambda_{1}+\lambda_{2}-2\lambda_{1}\lambda_{2}+\left(1-\lambda_{1}\right)\left(1-\lambda_{2}\right)\sum_{k=u}^{t-2}\frac{1}{k}\right)}^{2}]\sigma_{W}^{2},\;\text{for}\;t>2 \end{array}\right.$$ (14)

respectively. The expressions of the mean and variance of *HH*_*t*_ statistic are derived in S3 Appendix of this paper.

Using the above properties, the control limits for HHWMA *W* scheme are defined by

$$HUCL_{t}\;/HLCL_{t}=\mu_{W}\pm L_{HH}\sqrt{Var\left(HH_{t}\right)},$$ (15)

where *L*_*HH*_ (*L*_*HH*_ > 0) is the control limits constant of the HHWMA *W* scheme. The center line is equal to *μ*_*W*_ (i.e., *CL* = *μ*_*W*_). Thus, the HHWMA *W* scheme gives an OOC signal if the *HH*_*t*_ statistic plots beyond the control limits defined in Eq (15).

### 3. The operational procedure of the proposed HWMA, DHWMA and HHWMA *W* schemes

Most of the nonparametric monitoring schemes do not have specific or exact expressions of the run-length distribution. Hence, exact formulas as well as the Markov chain approach cannot be used in such a scenario. Therefore, Monte Carlo simulation approach is recommended since it can solve any complex problem through organised computer programs coding. In this paper, the proposed HWMA, DHWMA and HHWMA *W* schemes are constructed using the following steps:

**Step 1:** Generate a reference sample, *X*, of size *m* from an IC process, say *N*(0,1) distribution.
**Step 2:** Generate a test sample, *Y*, of size *n* independently from the reference sample. For the IC case, the distributions of the reference and test samples are identical (we say that *δ* = 0, thus *Y* ~ *N* (0,1)). For the OOC case, the distribution for the test sample is taken to be the same form as that for the reference sample, but with a shift in the location parameter in units of the population standard deviation (*δ* ≠ 0), in our example, *Y* ~ *N*(*δ*,1).
**Step 3:** The *W* statistic is calculated using Eq (1) as explained in Section 2.
**Step 4:** Calculate the mean and variance of the *W* statistic when the process is IC using Eq (2). These characteristics are used in Steps 5 and 6.
**Step 5:** (a) To construct the HWMA *W* scheme, we use the statistic defined in Eq (3).
    (b) To construct the DHWMA *W* scheme, we use the statistic defined in Eq (7).
    (c) To construct the HHWMA *W* scheme, we use the statistic defined in Eq (12).
**Step 6:** The control limits constants and the process design parameters are chosen such that the attained *ARL*_0_ is equal (or closer) to the nominal value of 500.
  (a) The time varying control limits of the HWMA *W* scheme are computed using Eq (6). The HWMA *W* scheme gives a signal if the charting statistic computed in Step 5(a) plots beyond the control limits calculated using Eq (6).
  (b) The control limits of the DHWMA *W* sheme are computed using Eq (11). The DHWMA *W* scheme gives a signal if the charting statistic computed in Step 5(b) plots beyond the control limits calculated using Eq (11).
  (c) The control limits of the HHWMA *W* sheme are computed using Eq (15). The HHWMA *W* scheme gives a signal if the charting statistic computed in Step 5(c) plots beyond the control limits calculated using Eq (15).

Note that other memory-type schemes based on the *W* statistic can also be constructed in a similar way.

## 4. IC and OOC performance analyses

### 4.1 Performance metrics

The characteristics of the run-length (*RL*) such as the average *RL* (*ARL*), the standard deviation of the *RL* (*SDRL*) as well as the expected *ARL* (*EARL*) and the expected *SDRL* (*ESDRL*) are the most popular metrics used to evaluate the performance of a monitoring scheme. The *RL* is the number of rational subgroups to be plotted on a scheme before it signals an OOC situation for the first time. Since the *RL* distribution is highly skewed, researchers advocate that the percentiles of the *RL* distribution provide more and meaningful information than the *ARL* value; see for example, [50]. It is very important to know that the *ARL* values measure the performance of a scheme for specific shifts. However, in practice, it is more crucial to investigate the performance of a monitoring scheme for a range of shifts, i.e., the overall performance for certain range of shifts. In this case, the SPM literature recommends the use of *EARL* or other expected characteristics of the *RL* to assess the overall performance of a scheme; see [51].

This paper uses Monte Carlo simulations in SAS ®9.4/IML15.4 with 20000 replications to assess the HWMA *W*, DHWMA *W* and HHWMA *W* schemes in terms of the above-mentioned metrics using different distributions. The distribution considered in this paper are the standard normal distribution (denoted as *N*(0,1)), Student *t* distribution with degrees of freedom 5 (denoted as *t*(5)) and gamma distribution with a shape parameter of 3 and a scale parameter of 1 (denoted as *GAM*(3,1)).

The *EARL* and *ESDRL* are mathematically defined as follows

$$EARL_{\left(\delta_{min},\delta_{max}\right]}=\frac{1}{\Delta }\sum_{\delta =\delta_{min}}^{\delta_{max}}ARL\left(\delta \right)$$ (16)

and

$$ESDRL_{\left(\delta_{min},\delta_{max}\right]}=\frac{1}{\Delta }\sum_{\delta =\delta_{min}}^{\delta_{max}}SDRL\left(\delta \right)$$ (17)

where *ARL*(*δ*) and *SDRL*(*δ*) are the values of the *ARL* and *SDRL* for a specific shift *δ* in standard deviation unit and Δ is the number of increments between the lower and upper bound shifts (i.e., *δ*_*min*_ and *δ*_*max*_). In this paper, we use increments equal to 0.1.

### 4.2 Performance analysis

In this section, we study the sensitivity (or performance) of the proposed HWMA, DHWMA and HHWMA *W* schemes under the *N*(0,1), *t*(5) and *GAM*(3,1) distributions.

#### 4.2.1 Performance of the proposed HWMA *W* scheme

Table 2 presents the IC and OOC *ARL* profiles of the proposed HWMA *W* scheme under *N*(0,1), *t*(5) and *GAM*(3,1) distributions when *n* = 5, *m* = 100, λ ∈{0.05,0.25,0.5} for a nominal IC *ARL* (*ARL*_0_) of 500. From Table 2, it can be seen that when *λ* = 0.05, the proposed HWMA *W* scheme yields the attained *ARL*_0_ values of 502.47, 499.37 and 504.89 under the *N*(0,1), *t*(5) and *GAM*(3,1) distributions, respectively. It can be observed that these attained *ARL*_0_ values are much closer to the nominal *ARL*_0_ value of 500, i.e. within the 10% of the desired nominal *ARL*_0_ value as suggested by the SPM literature; see for example [33]. Other values of the attained *ARL*_0_ values are also much closer to the nominal *ARL*_0_ value of 500. Therefore, it can be concluded that the HWMA *W* scheme is IC robust which means that the characteristics of the IC *RL* are much closer to 500 across all continuous probability distributions.

**Table 2:** The *ARL* profile of the HWMA *W* scheme when (*m*,*n*) = (100,5) and ***λ*** ∈ {0.05, 0.25, 0.5} for a nominal *ARL*_0_ = 500 under *N*(0,1), *t*(5) and *GAM*(3,1) distributions.

	*N*(0,1)			*t*(5)			*GAM*(3,1)		
Shift (*δ*)	*λ* = 0.05	*λ* = 0.25	*λ* = 0.5	*λ* = 0.05	*λ* = 0.25	*λ* = 0.5	*λ* = 0.05	*λ* = 0.25	*λ* = 0.5
0.0	502.47	498.21	502.04	499.37	504.68	503.48	504.89	501.14	506.14
0.1	338.73	373.93	380.88	261.15	310.07	372.76	336.74	437.58	421.30
0.2	119.08	137.51	174.03	86.45	97.00	96.07	105.27	160.44	203.76
0.3	37.46	37.80	43.29	27.67	27.45	47.17	29.82	40.58	56.90
0.4	16.02	15.68	26.83	13.71	12.11	18.27	13.04	13.73	26.54
0.5	10.30	9.78	13.77	8.26	7.58	9.78	8.42	8.17	12.58
0.6	7.53	6.77	8.44	6.81	6.47	6.32	6.26	5.75	7.63
0.7	5.84	5.28	5.90	5.40	4.32	4.46	4.97	4.49	5.16
0.8	4.86	4.26	4.40	4.16	3.60	3.51	4.22	3.66	3.80
0.9	4.13	3.56	3.48	3.61	3.08	2.84	3.69	3.16	3.04
1.0	3.62	3.09	2.92	3.16	2.71	2.44	3.29	2.78	2.56
1.1	3.23	2.75	2.50	2.86	2.41	2.16	3.01	2.51	2.22
1.2	2.94	2.46	2.21	2.58	2.16	1.91	2.78	2.28	1.99
1.3	2.67	2.25	1.97	2.35	1.97	1.74	2.54	2.07	1.81
1.4	2.43	2.04	1.81	2.14	1.81	1.61	2.33	1.88	1.68
1.5	2.23	1.87	1.66	1.94	1.67	1.49	2.12	1.71	1.41
***EARL***_**(0,0.7]**_	76.42	83.82	93.31	58.49	66.43	79.26	72.07	95.82	104.84
***EARL***_**(0.7,1.5]**_	3.26	2.79	2.62	2.85	2.43	2.21	3.00	2.51	2.31
***EARL***_**(0,1.5]**_	37.40	40.60	44.94	28.82	32.29	38.17	35.23	46.05	50.16
***L***_***H***_	2.9567	2.9559	2.8699	2.9567	2.9559	2.8699	2.9567	2.9559	2.8699

In terms of the OOC *ARL* profile, as the value of *λ* increases, the width of the control limits decreases; however, the sensitivity of the HWMA *W* decreases. For small shifts in the process mean, the sensitivity of the HWMA *W* scheme decreases as *λ* increases. However, the converse is true for moderate shifts. For instance, under the *N*(0,1) distribution, when *λ* = 0.05, 0.25 and 0.5, the *EARL*_(0.7,1.5]_ is found to be equal to 3.26, 2.79 and 2.62, respectively. This shows that for moderate shifts in the process mean, there is an increase in the sensitivity of the HWMA *W* scheme as *λ* increases. For small-to-moderate as well as from small-to-large shifts, the smaller the value of *λ*, the higher the sensitivity of the proposed HWMA *W* scheme. From Table 2, it can also be seen that for small values of *λ*, i.e., 0 < *λ* < 0.25, and small shifts in the process mean, the proposed HWMA *W* scheme performs better under heavy-tailed distributions followed by skewed distributions. For instance, when *λ* = 0.05, the HWMA *W* scheme yields *EARL*_(0,0.7]_ values of 76.2, 58.49 and 72.07 under the *N*(0,1), *t*(5) and *GAM*(3,1) distributions, respectively. However, when 0.25 ≤ *λ* < 1, the proposed HWMA *W* scheme performs better under heavy-tailed distributions followed by symmetric distributions. For instance, when *λ* = 0.5, the HWMA scheme yields *EARL*_(0,0.7]_ values of 93.31, 79.26 and 104.84 under the *N*(0,1), *t*(5) and *GAM*(3,1) distributions, respectively. This conclusion is also true in terms of the overall performance when *δ* ∈ (0,1.5]; see Table 2 performance in terms of the *EARL*_(0,1.5]_. For moderate shifts in the process mean, regardless of the magnitude of *λ*, the proposed HWMA *W* scheme performs better under heavy-tailed distributions followed by skewed distributions. For instance, when *λ* = (0.05, 0.25, 0.5), the HWMA *W* scheme yields *EARL*_(0.7,1.5]_ values of (3.26, 2.79, 2.62), (2.85, 2.43, 2.21) and (3.00, 2.51, 2.31) under the *N*(0,1), *t*(5) and *GAM*(3,1) distributions, respectively.

**Fig 1:**
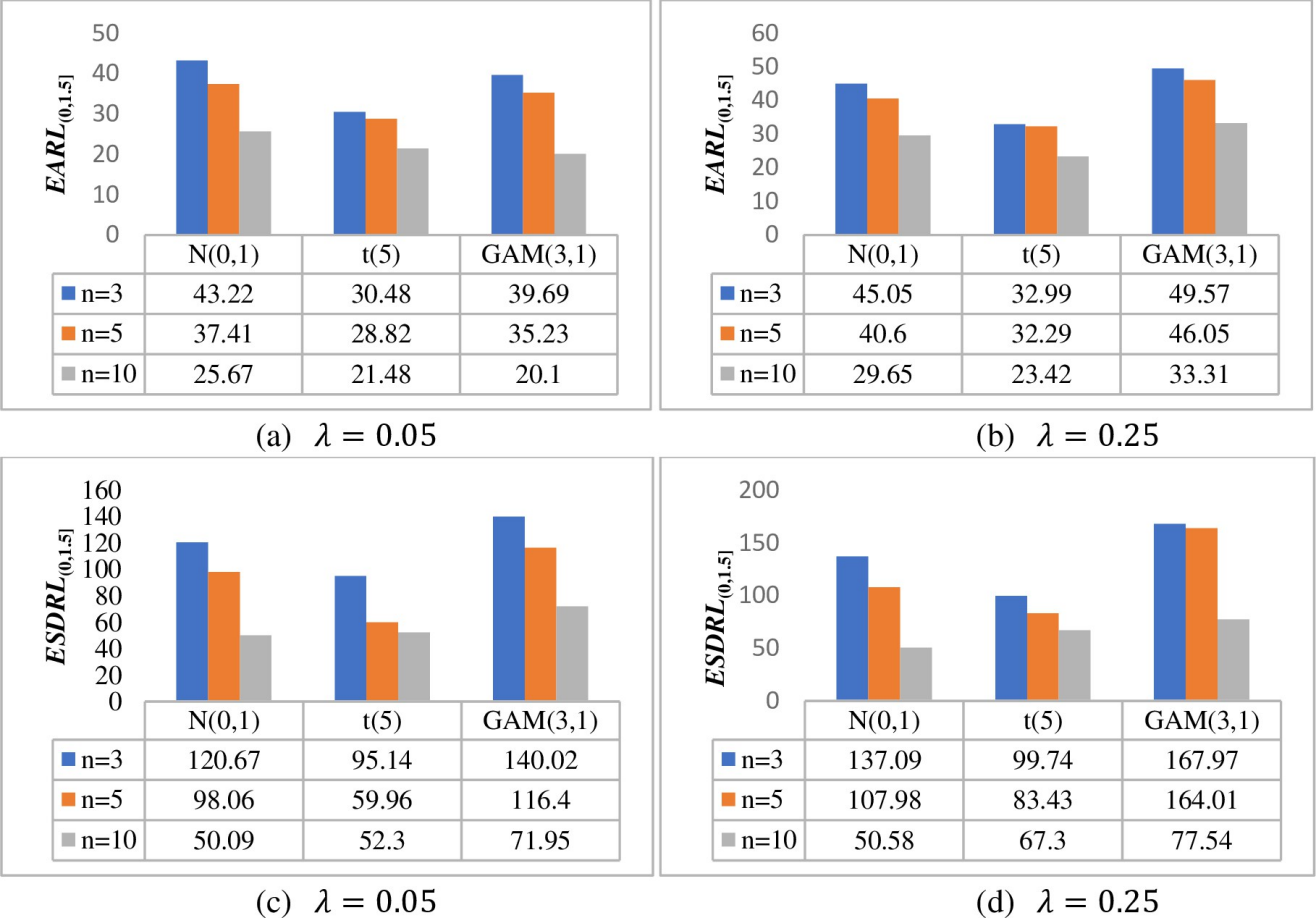
Effect of the phase II sample size on the overall performance of the HWMA *W* scheme when *m* = 100, *λ* ∈ {0.05, 0.25} and *n* ∈ {3, 5, 10} under *N*(0,1), *t*(5) and *GAM*(3,1) distributions.

Fig 1 investigates the effect of the test sample (i.e. phase II) on the overall performance of the HWMA *W* scheme in terms of the *EARL*_(0,1.5]_ and *ESDRL*_(0,1.5]_ when *m* = 100, *n* ∈{3,5,10} and *λ* ∈{0.05, 0.25} for a nominal *ARL*_0_ value of 500. Thus, it can be observed that the larger the test sample, the more sensitive the proposed scheme is. For instance, when *λ* = 0.05, under the *N*(0,1) distribution, the HWMA *W* scheme yields *EARL* values of 43.22, 37.41 and 25.67 when *n* = 3, 5 and 10, respectively (see Fig 1A). The findings remain the same for other values of *λ* across all continuous probability distributions; see also Fig 1B). The same conclusion is also deduced in terms of the *ESDRL* profile (see Fig 1C and 1D).

**Fig 2:**
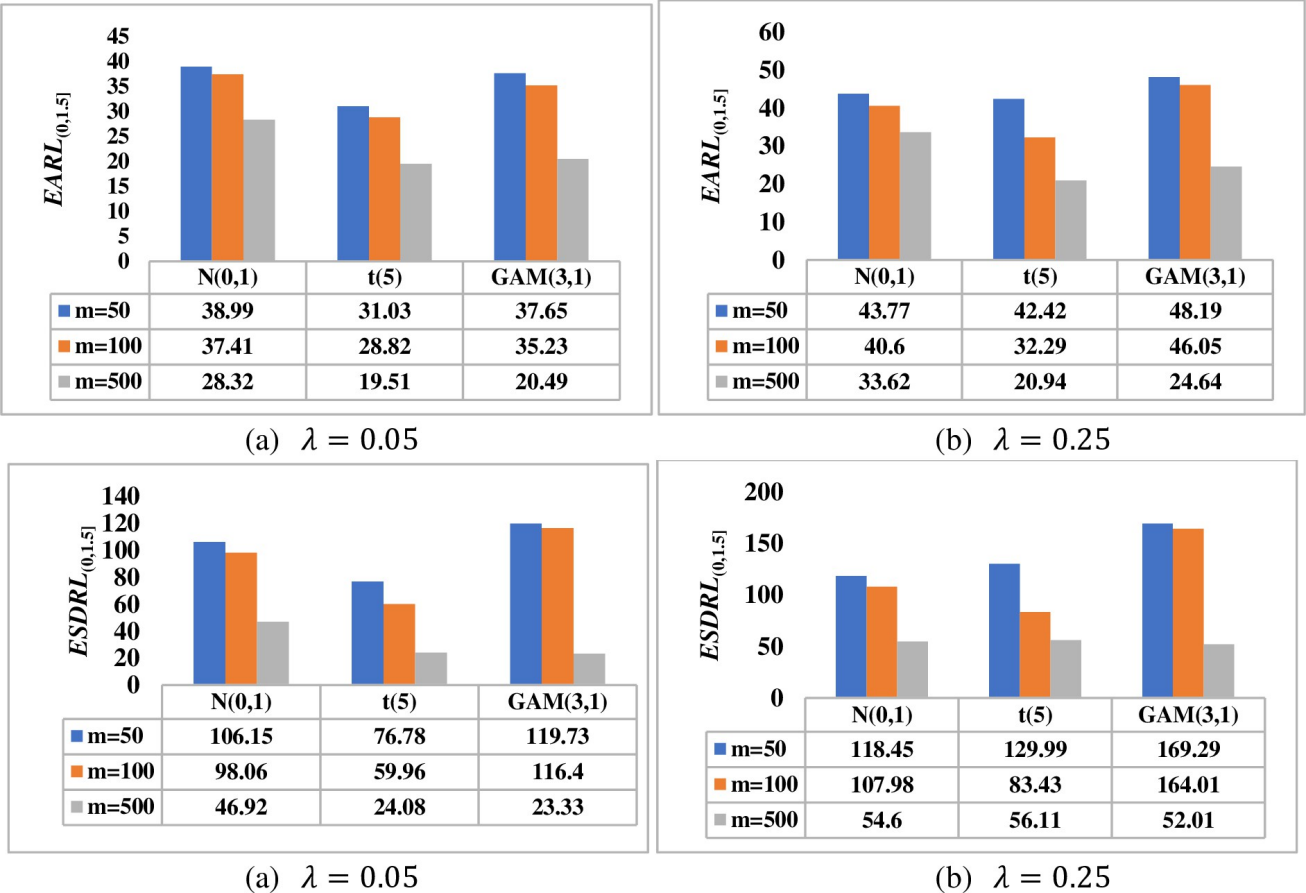
Effect of the phase I sample size on the performance of HWMA *W* scheme when *n* = 5, *λ* ∈ {0.05, 0.25} and *m* ∈ {50, 100,500} under *N*(0,1), *t*(5) and *GAM*(3,1) distributions.

Fig 2 investigates the effect of the reference sample (i.e. phase I) on the overall performance of the HWMA *W* scheme in terms of the *EARL*_(0,1.5]_ and *ESDRL*_(0,1.5]_ when *m* ∈ {50,100,500}, *n* = 5 and *λ* ∈{0.05, 0.25} for a nominal *ARL*_0_ value of 500. Thus, it can be observed that the larger the phase I sample size, the more sensitive the HWMA *W* scheme is. The smaller the phase I sample size, the higher the variability in the *RL* distribution. For instance, when *λ* = 0.25, under the *t*(5) distribution, the HWMA *W* scheme yields *ESDRL* values of 129.99, 83.43 and 56.11 when *m* = 50, 100 and 500, respectively (see Fig 2B. The findings remain the same for other values of *λ*.

#### 4.2.2 Performance of the proposed DHWMA *W* scheme

Table 3 displays the IC and OOC *ARL* profiles of the proposed DHWMA *W* scheme under *N*(0,1), *t*(5) and *GAM*(3,1) distributions when *n* = 5, *m* = 100, *λ* ∈{0.05,0.25,0.5} for a nominal *ARL*_0_ value of 500. From Table 3, it can be seen that when *λ* = 0.05, 0.25 and 0.5, the proposed DHWMA *W* scheme yields attained *ARL*_0_ values of (499.39, 502.63, 503.37), (495.43, 512.80, 512.27) and (508.70, 499.08, 501.13) under the *N*(0,1), *t*(5) and *GAM*(3,1) distributions, respectively. It can be observed these attained *ARL*_0_ values are much closer to the nominal *ARL*_0_ value of 500. Therefore, it can be concluded that the DHWMA *W* scheme is IC robust.

**Table 3:** The *ARL* profile of the DHWMA *W* scheme when (*m*,*n*) = (100,5) and *λ* ∈ {0.05, 0.25, 0.5} for a nominal *ARL*_0_ = 500 under *N*(0,1), *t*(5) and *GAM*(3,1) distributions.

	*N*(0,1)			*t*(5)			*GAM*(3,1)		
Shift (*δ*)	*λ* = 0.05	*λ* = 0.25	*λ* = 0.5	*λ* = 0.05	*λ* = 0.25	*λ* = 0.5	*λ* = 0.05	*λ* = 0.25	*λ* = 0.5
0.0	499.39	502.63	503.37	495.43	512.80	512.27	508.70	499.08	501.13
0.1	254.17	262.48	367.40	200.09	236.70	333.39	333.84	410.44	539.19
0.2	73.12	78.22	166.25	103.53	156.47	122.50	107.62	163.98	148.82
0.3	30.38	44.26	30.79	21.89	21.97	26.01	42.26	26.49	21.82
0.4	19.06	17.05	19.39	14.51	15.80	11.69	15.60	12.20	14.25
0.5	11.45	11.58	11.74	7.28	8.60	7.47	10.81	8.79	9.24
0.6	8.78	8.36	7.89	6.36	7.29	5.92	6.59	5.93	6.08
0.7	6.00	6.55	4.98	4.43	4.98	4.55	5.36	4.57	4.95
0.8	5.03	5.19	4.39	3.33	3.76	3.69	4.19	3.95	3.82
0.9	4.43	4.16	3.56	2.93	3.73	3.09	3.41	3.11	3.39
1.0	3.75	3.42	3.38	2.88	2.95	2.94	3.04	2.77	2.78
1.1	2.89	3.19	2.59	2.78	2.76	2.45	2.42	2.43	2.56
1.2	2.29	2.50	2.43	2.51	2.45	2.11	1.94	2.25	2.21
1.3	2.00	2.20	2.32	2.24	2.01	1.95	1.85	2.04	2.05
1.4	1.85	2.00	2.04	1.56	1.91	1.89	1.41	1.79	1.99
1.5	1.51	1.94	1.85	1.03	1.62	1.55	1.29	1.76	1.70
***EARL***_**(0,0.7]**_	57.57	61.21	86.92	51.16	64.54	73.08	74.58	90.34	106.34
***EARL***_**(0.7,1.5]**_	2.97	3.08	2.82	2.41	2.65	2.46	2.44	2.51	2.56
***EARL***_**(0,1.5]**_	28.45	30.21	42.07	25.16	31.53	35.41	36.11	43.5	50.99
***L***_***DH***_	1.2959	2.7999	2.9995	1.2959	2.7999	2.9995	1.2959	2.7999	2.9995

In terms of the OOC *ARL* profile, as the value of *λ* increases, the width of the control limits increases as well; however, the sensitivity of the DHWMA *W* decreases. For small shifts in the process mean, the sensitivity of the DHWMA *W* scheme decreases as *λ* increases. However, the converse is true for moderate shifts under symmetric and heavy-tailed distribution. For instance, under the *t*(5) distribution, when *λ* = 0.05, 0.25 and 0.5, the *EARL*_(0.7,1.5]_ is found to be equal to 2.41, 2.65 and 2.46, respectively. For small-to-moderate as well as from small-to-large shifts, the smaller the value of *λ*, the higher the sensitivity of the proposed DHWMA *W* scheme. From Table 3, it can also be seen that for small shifts in the process mean, the proposed DHWMA *W* scheme performs better under heavy-tailed distributions followed by symmetric distributions. For instance, when *λ* = 0.05, the DHWMA scheme yields *EARL*_(0,0.7]_ values of 57.57, 51.16 and 74.58 under the *N*(0,1), *t*(5) and *GAM*(3,1) distributions, respectively. However, for moderate shifts in the process mean, regardless of the magnitude of *λ*, the proposed DHWMA *W* scheme performs better under heavy-tailed distributions followed by skewed distributions. For instance, when *λ* **=** 0.25, the DHWMA scheme yields *EARL*_(0.7,1.5]_ values of 3.08, 2.65 and 2.51 under the *N*(0,1), *t*(5) and *GAM*(3,1) distributions, respectively. This conclusion is also true in terms of the overall performance when *δ* ∈ (0,1.5]; see Table 3 in terms of the *EARL*_(0,1.5]_ values.

**Fig 3:**
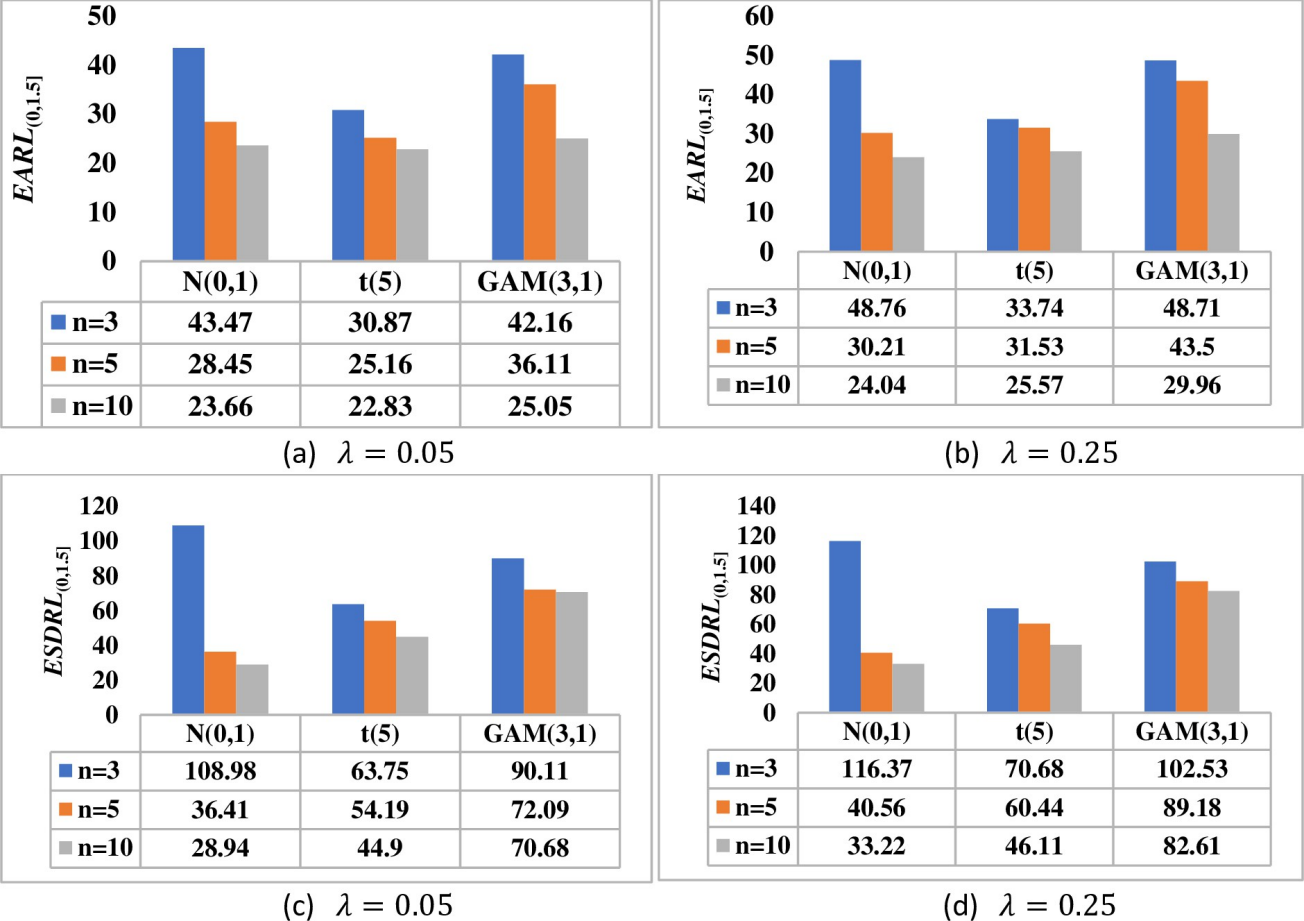
Effect of the phase II sample size on the performance of DHWMA *W* scheme when *m* = 100, *λ* ∈ {0.05, 0.25} and *n* ∈ {3, 5, 10} under *N*(0,1), *t*(5) and *GAM*(3,1) distributions.

**Fig 4:**
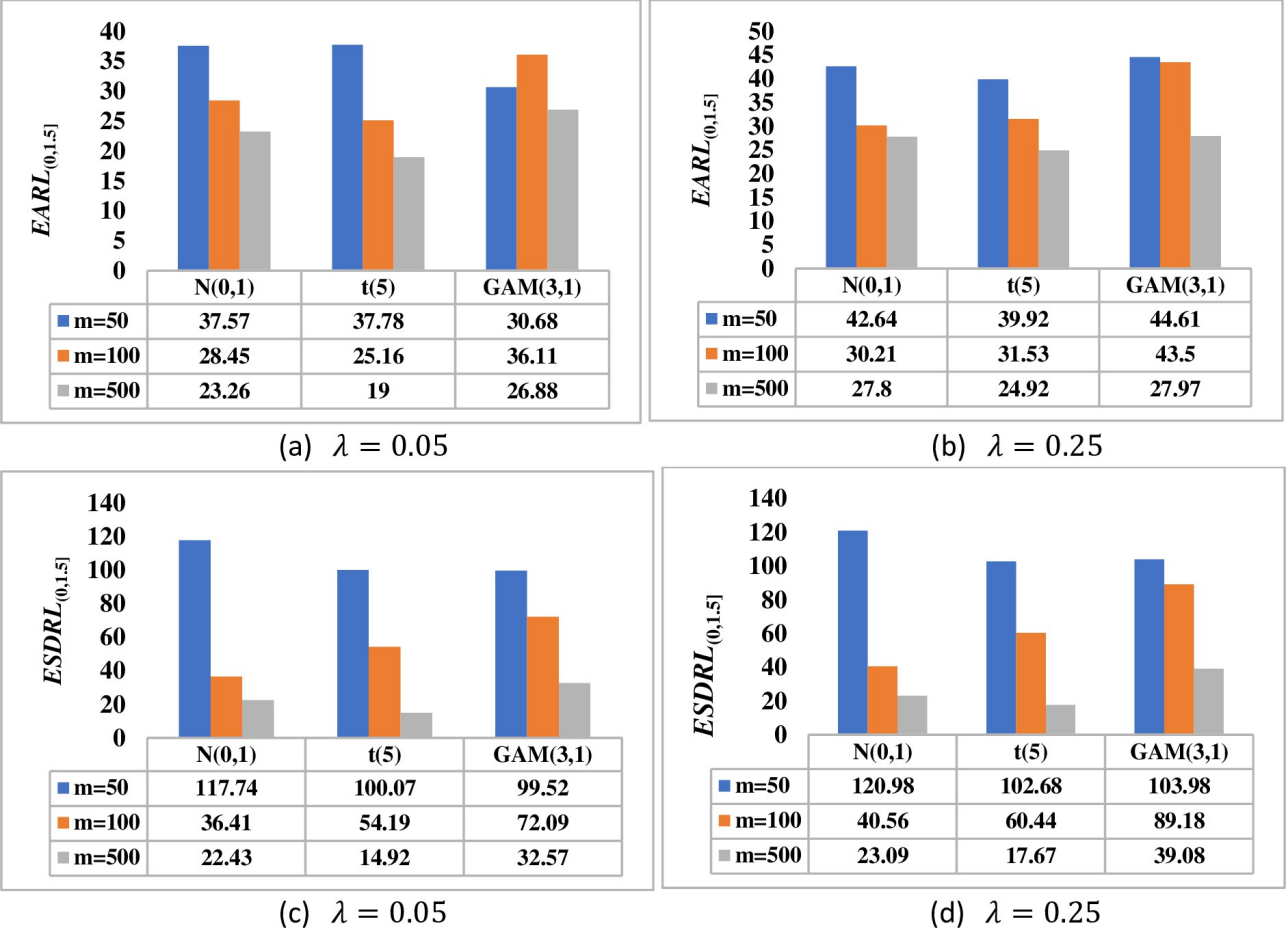
Effect of the phase I sample size on the performance of DHWMA *W* scheme when *n* = 5, *λ* ∈ {0.05, 0.25} and *m* ∈ {50, 100,500} under *N*(0,1), *t*(5) and *GAM*(3,1) distributions.

Fig 3 shows that as the phase II sample size *n* increases, the better the proposed DHWMA *W* scheme becomes in detecting shifts in the process mean. Note that as the value of *λ* increases, the proposed scheme becomes less sensitive. This is revealed by larger *EARL* and *ESDRL* values for large values of *λ* and small *EARL* and *ESDRL* values for small values of *λ*. Fig 4 shows that the larger the phase I sample size *m*, the more sensitive the DHWMA *W* scheme becomes–except for cases where *λ* is small under skewed distributions, see Fig 4A. As *λ* increases, Fig 4 also reveals that the DHWMA *W* scheme becomes less sensitive since the *EARL* and *ESDRL* values get larger. Note that while increasing *m* and/or *n* helps to increase the sensitivity of the proposed scheme, it also increases the costs of inspection and implementation; moreover, large samples are not easy to find. Therefore, a more reasonable sample size must be chosen in order to strike a balance between the sensitivity of the proposed scheme and the cost related to its implementation.

#### 4.2.3 Performance of the proposed HHWMA *W* scheme

Table 4 displays the IC and OOC *ARL* profiles of the proposed HHWMA *W* scheme under *N*(0,1), *t*(5) and *GAM*(3,1) distributions when *n* = 5, *m* = 100, *λ*_1_ ∈{0.1, 0.2} and *λ*_2_ ∈{0.05,0.25,0.5} for a nominal *ARL*_0_ = 500. From Table 4, it can be seen that when *λ*_1_ = 0.1 and *λ*_2_ = 0.05, 0.25 and 0.5, the proposed HHWMA *W* scheme yields attained *ARL*_0_ values of (498.09, 502.89, 498.16), (494.78, 497.08, 508.29) and (509.48, 498.9, 496.86) under the *N*(0,1), *t*(5) and *GAM*(3,1) distributions, respectively. Also, it can be seen that when *λ*_1_ = 0.2 and *λ*_2_ = 0.05, 0.25 and 0.5, the proposed HHWMA *W* scheme yields attained *ARL*_0_ values of (503.16, 506.08, 499.80), (502.44, 493.90, 499.70) and (502.86, 502.90, 505.40) under the *N*(0,1), *t*(5) and *GAM*(3,1) distributions, respectively. Thus, it can be observed that in both cases the attained *ARL*_0_ values are much closer to the nominal *ARL*_0_ value of 500 across different continuous probability distributions. Therefore, it can be concluded that the HHWMA *W* scheme is IC robust.

**Table 4:** The *ARL* profile of the HHWMA *W* scheme when (*m*,*n*) = (100,5), *λ*_1_ ∈ {0.1,0.2} and *λ*_2_ ∈ {0.05,0.25,0.5} for a nominal *ARL*_0_ = 500 under *N*(0,1), *t*(5) and *GAM*(3,1) distributions.

	*λ*_1_ = 0.1									*λ*_1_ = 0.2								
	*N*(0,1)			*t*(5)			*GAM*(3,1)			*N*(0,1)			*t*(5)			*GAM*(3,1)		
Shift (*δ*)	*λ*_2_ = 0.05	*λ*_2_ = 0.25	*λ*_2_ = 0.5	*λ*_2_ = 0.05	*λ*_2_ = 0.25	*λ*_2_ = 0.5	*λ*_2_ = 0.05	*λ*_2_ = 0.25	*λ*_2_ = 0.5	*λ*_2_ = 0.05	*λ*_2_ = 0.25	*λ*_2_ = 0.5	*λ*_2_ = 0.05	*λ*_2_ = 0.25	*λ*_2_ = 0.5	*λ*_2_ = 0.05	*λ*_2_ = 0.25	*λ*_2_ = 0.5
0.0	498.09	502.89	498.16	494.78	497.08	508.29	509.48	498.90	496.86	503.16	506.08	499.81	502.44	493.90	499.77	502.86	502.90	505.49
0.1	337.45	346.02	344.17	260.97	203.87	275.89	297.91	323.90	314.94	340.05	365.33	352.23	235.70	308.97	301.67	249.25	331.29	374.65
0.2	100.53	154.18	167.13	66.69	129.32	142.48	75.54	95.19	109.49	55.40	156.15	155.45	91.31	86.87	105.26	122.10	113.51	73.10
0.3	18.65	26.83	23.78	15.05	20.73	13.91	15.12	22.10	13.12	29.65	19.67	41.91	23.29	12.62	27.31	19.71	22.15	26.31
0.4	14.99	15.42	12.85	10.6	11.68	7.22	9.28	9.20	7.48	11.59	8.79	16.10	8.97	8.80	16.61	8.81	9.60	12.39
0.5	6.50	7.47	6.09	5.92	7.75	4.44	5.82	5.70	4.32	5.98	5.86	12.36	6.39	5.20	9.48	6.17	4.76	9.05
0.6	5.52	6.84	4.54	4.40	5.18	3.42	4.40	4.16	2.80	4.56	4.53	7.80	5.03	4.07	7.14	4.56	3.10	6.52
0.7	4.15	5.09	2.79	3.93	4.05	2.32	3.92	3.30	2.07	3.15	3.79	5.99	3.80	2.99	5.62	3.70	2.76	5.48
0.8	3.54	4.16	2.35	3.35	3.42	2.04	3.44	2.70	1.99	2.37	3.38	4.73	3.42	1.95	4.54	3.32	2.29	4.19
0.9	3.48	3.41	2.18	3.12	2.59	1.74	3.28	2.30	1.31	1.98	3.08	4.23	3.07	1.67	3.45	3.17	1.87	3.70
1.0	2.98	2.77	1.85	2.94	2.23	1.36	3.00	2.00	1.30	1.64	2.92	3.67	2.65	1.61	2.79	2.97	1.56	3.06
1.1	2.86	2.54	1.58	2.58	2.11	1.31	2.76	1.70	1.20	1.60	2.65	2.96	2.47	1.51	2.67	2.81	1.23	2.64
1.2	2.80	2.26	1.53	2.46	1.96	1.29	2.71	1.50	1.06	1.33	2.48	2.78	2.39	1.45	2.28	2.68	1.11	2.41
1.3	2.48	2.17	1.32	2.36	1.61	1.19	2.55	1.30	1.03	1.20	2.32	2.44	2.12	1.25	2.01	2.53	1.02	2.04
1.4	2.36	1.67	1.22	2.17	1.50	1.13	2.34	1.20	1.01	1.14	2.23	2.28	2.06	1.05	1.83	2.41	1.01	1.94
1.5	2.03	1.50	1.08	2.03	1.42	1.06	2.15	1.10	1.00	1.03	1.95	1.93	1.81	1.04	1.73	2.19	1.00	1.72
***EARL***_**(0,0.7]**_	69.68	80.26	80.19	52.51	54.65	64.24	58.86	66.22	64.89	64.34	80.59	84.55	53.50	61.36	67.58	59.19	69.60	72.50
***EARL***_**(0.7,1.5]**_	2.82	2.56	1.64	2.63	2.11	1.39	2.78	1.73	1.24	1.54	2.63	3.13	2.50	1.44	2.66	2.76	1.39	2.71
***EARL***_**(0,1.5]**_	34.02	38.82	38.30	25.90	26.63	30.72	28.95	31.82	30.94	30.84	39.01	41.12	26.30	29.40	32.96	29.09	33.22	35.28
***L***_***HH***_	1.3280	2.5010	3.1100	1.3280	2.5010	3.1100	1.3280	2.5010	3.1100	1.4695	2.7999	3.0795	1.4695	2.7999	3.0795	1.4695	2.7999	3.0795

Since the proposed HHWMA *W* scheme is confirmed to be IC robust, it is now fair enough to evaluate its performance under different distributions. When *λ*_1_ is kept constant, as *λ*_2_ increases, the width of the control limits get wider; however, the sensitivity of the HHWMA *W* scheme in terms of the *ARL* values decreases. For small shifts in the process mean, the sensitivity of the HHWMA *W* scheme decreases as *λ*_2_ increases. However, the converse is true for moderate shifts. For instance, under the *t*(5) distribution, when *λ*_1_ = 0.1 and *λ*_2_ = 0.05, 0.25 and 0.5, for small shifts, the *EARL*_(0,0.7]_ is equal to 52.51, 54.65 and 64.24, respectively; whereas, for moderate shifts, the *EARL*_(0.7,1.5]_ is equal to 2.63, 2.11 and 1.39, respectively. This shows that for moderate shifts in the process mean, there is an increase in the sensitivity of the HHWMA *W* scheme as *λ*_2_ increases. For small-to-moderate as well as from small-to-large shifts, the smaller the value of *λ*_2_, the higher the sensitivity of the proposed HHWMA *W* scheme. From Table 4, it can also be seen that for small shifts in the process mean, the proposed HHWMA *W* scheme performs better under heavy-tailed distributions followed by skewed distributions. For instance, when *λ*_1_ = 0.1 and *λ*_2_ = 0.05, the HHWMA scheme yields *EARL*_(0,0.7]_ values of 69.68, 52.51 and 58.86 under the *N*(0,1), *t*(5) and *GAM*(3,1) distributions, respectively. However, for moderate shifts in the process mean, regardless of the magnitude of *λ*_2_, the proposed HHWMA *W* scheme performs better under skewed distributions followed by heavy-tailed distributions when *λ*_1_ is kept small. For instance, when *λ*_1_ = 0.1 and *λ*_2_ = 0.25, the HHWMA *W* scheme yields *EARL*_(0.7,1.5]_ values of 2.56, 2.11 and 1.73 under the *N*(0,1), *t*(5) and *GAM*(3,1) distributions, respectively. However, as *λ*_1_ increases, the HHWMA *W* scheme performs better under heavy-tailed distributions followed by symmetric ones. This conclusion is also true in terms of the overall performance when *δ* ∈ (0,1.5] regardless of the values of *λ*_1_ and *λ*_2_; see Table 4 performance in terms of the *EARL*_(0,1.5]_ values.

**Fig 5:**
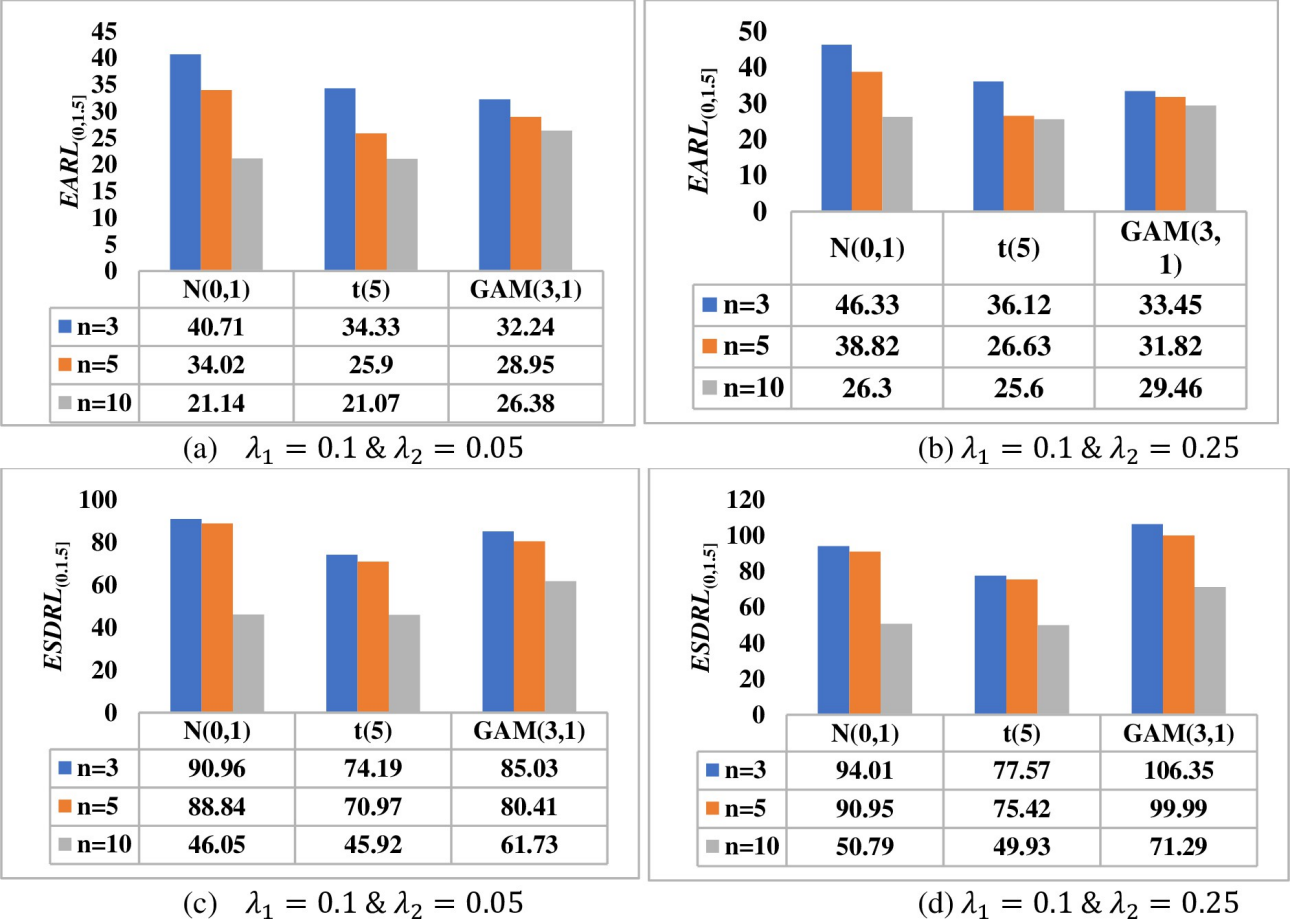
Effect of the phase II sample size on the performance of HHWMA *W* scheme when *m* = 100, *λ*_1_ = 0.1, *λ*_2_ ∈ {0.05, 0.25} and *n* ∈ {3, 5, 10} under *N*(0,1), *t*(5) and *GAM*(3,1) distributions.

**Fig 6:**
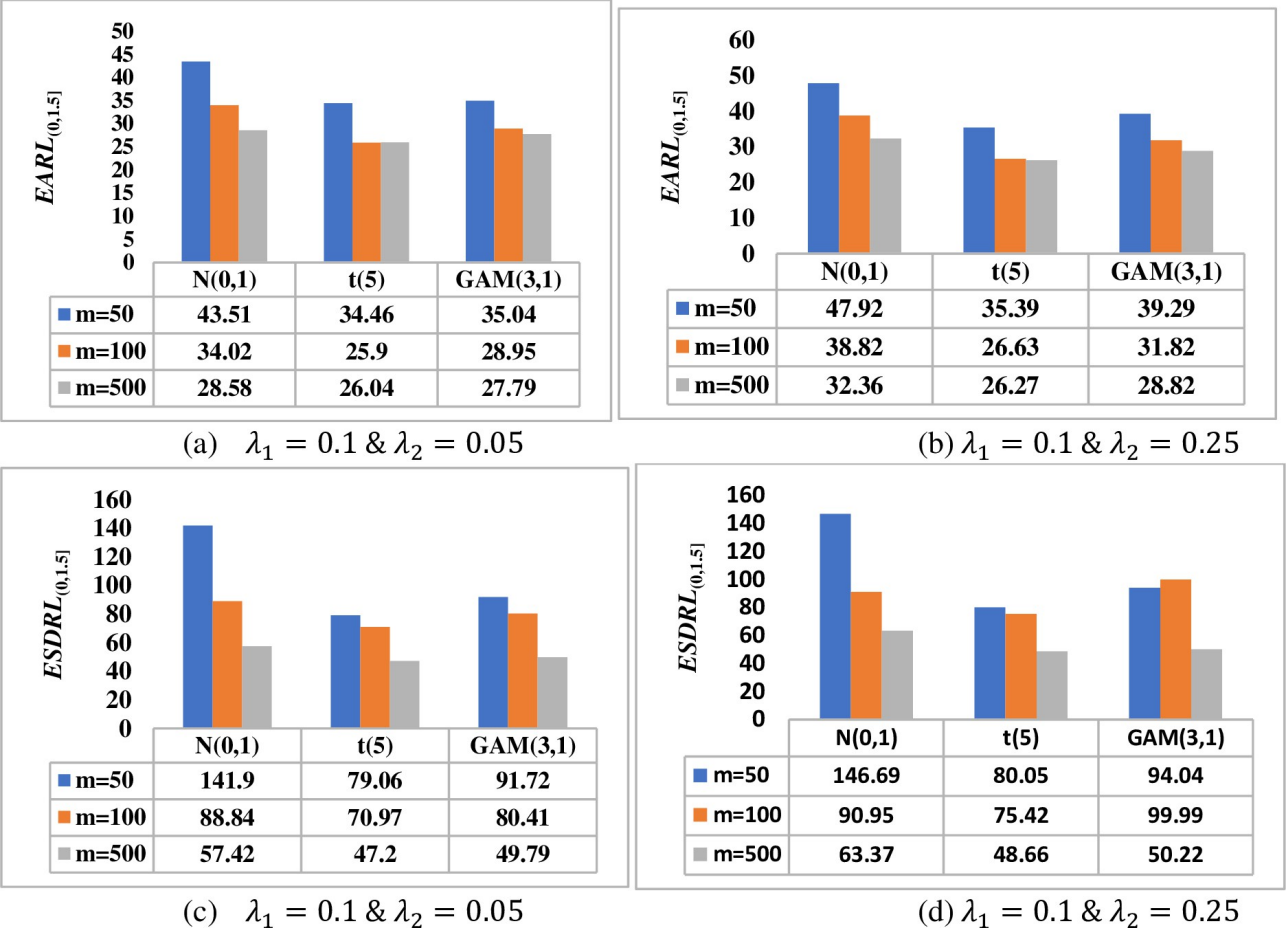
Effect of the phase I sample size on the performance of HHWMA *W* scheme when *n* = 5, *λ*_1_ = 0.1, *λ*_2_ ∈ {0.05, 0.25} and *m* ∈ {50, 100,500} under *N*(0,1), *t*(5) and *GAM*(3,1) distributions.

Fig 5 shows that as the phase II sample size increases, the more sensitive the proposed HHWMA *W* scheme becomes in detecting shifts in the process mean. For a given value of *λ*_1_, as the value of *λ*_2_ increases, the proposed scheme becomes less sensitive. This is revealed by larger *EARL* and *ESDRL* values for large values of *λ*_2_ and smaller *EARL* and *ESDRL* values for small values of *λ*_2_. Fig 6 shows that the larger the phase I sample size *m*, the more sensitive the HHWMA *W* scheme becomes. Fig 6 also reveals that the HHWMA *W* scheme becomes less sensitive as *λ*_2_ increases. For instance, under the *N*(0,1) distribution, when *m* = 500, *λ*_1_ = 0.1, *λ*_2_ = 0.05 and 0.25, the *EARL*_(0,1.5]_ = 57.42 and 63.37, respectively. A similar pattern is observed in terms of the *ESDRL* values.

### 4.3 Performance comparison study

In this section, the proposed HWMA, DHWMA and HHWMA *W* schemes are compared to some existing memory-type monitoring schemes competitors, that is, the CUSUM and EWMA *W* schemes of [5] and the DEWMA *W* scheme of [49]. The comparison is made when *m* = 100, *n* = 5 and *λ* = 0.5 and for HHWMA *W* (*λ*_1_,_2_) = (0.1, 0.5) with shifts increasing by 0.1 from 0.1 to 1.5 under *N*(0,1), *t*(5) and *GAM*(3,1) distributions. Tables 5 to 7 present the OOC *ARL* performances of the competing schemes for specific shifts and overall performances along with their corresponding control limits constants. To study the overall performance of the competing charts for small, moderate and large shifts, the *EARL* values were calculated using Eq (16) where *EARL*_(0,0.7]_, *EARL*_(0.7,1.5]_ and *EARL*_(0,1.5]_ values correspond to small, moderate and small-to-moderate shifts, respectively. In Tables 5–7, the *EARL* results of the most effective scheme are boldfaced.

**Table 5:** The OOC *ARL* profiles of the proposed HWMA, DHWMA and HHWMA *W* schemes against the CUSUM, EWMA and DEWMA *W* schemes when (*m*,*n*,*λ*) = (100,5,0.5) for a nominal *ARL*_0_ = 500 under *N*(0,1) distribution.

Shift (*δ*)	CUSUM *W*	EWMA *W*	DEWMA *W*	HWMA *W*	DHWMA *W*	HHWMA *W*
0.1	400.24	381.13	372.74	380.88	367.40	324.17
0.2	184.22	177.30	174.88	174.03	166.25	187.13
0.3	69.29	66.60	63.22	43.29	30.79	23.78
0.4	27.71	26.44	26.49	26.83	19.39	12.85
0.5	15.33	13.91	13.82	13.77	11.74	6.09
0.6	5.09	7.45	7.27	8.44	7.89	4.54
0.7	4.34	6.03	5.90	5.90	4.98	2.79
0.8	4.00	5.52	4.66	4.40	4.39	2.35
0.9	3.11	3.68	3.41	3.48	3.56	2.18
1.0	3.64	3.42	3.13	2.92	3.38	1.85
1.1	3.18	2.80	2.80	2.50	2.59	1.58
1.2	3.07	2.67	2.54	2.21	2.43	1.53
1.3	2.46	2.42	2.18	1.97	2.32	1.32
1.4	1.34	2.19	2.00	1.81	2.04	1.22
1.5	1.10	2.00	1.92	1.66	1.85	1.08
***EARL***_**(0,0.7]**_	100.89	96.98	94.90	93.31	86.92	**80.19**
***EARL***_**(0.7,1.5]**_	2.74	3.09	2.83	2.62	2.82	**1.64**
***EARL***_**(0,1.5]**_	48.54	46.90	45.80	44.94	42.07	**38.30**
***L***	3.3890	3.3800	3.1999	2.8699	2.9995	3.1100

**Table 6:** The OOC *ARL* profiles of the proposed HWMA, DHWMA and HHWMA *W* schemes against the CUSUM, EWMA and DEWMA *W* schemes when (*m*,*n*,*λ*) = (100,5,0.5) for a nominal *ARL*_0_ = 500 under *t*(5) distribution.

Shift (*δ*)	CUSUM *W*	EWMA *W*	DEWMA *W*	HWMA *W*	DHWMA *W*	HHWMA *W*
0.1	384.19	381.40	362.72	372.76	333.39	275.89
0.2	187.41	139.74	137.85	96.07	122.50	142.48
0.3	55.33	51.18	45.10	47.17	26.01	13.91
0.4	19.37	18.52	18.42	18.27	11.69	7.22
0.5	9.65	9.20	8.88	9.78	7.47	4.44
0.6	7.82	7.64	7.37	6.32	5.92	3.42
0.7	5.45	5.55	5.50	4.46	4.55	2.32
0.8	4.34	3.90	3.76	3.51	3.69	2.04
0.9	3.91	3.61	3.42	2.84	3.09	1.74
1.0	3.36	3.14	2.91	2.44	2.94	1.36
1.1	3.11	2.77	2.60	2.16	2.45	1.31
1.2	2.88	2.52	2.33	1.91	2.11	1.29
1.3	2.52	2.20	2.00	1.74	1.95	1.19
1.4	2.30	1.89	1.75	1.61	1.89	1.13
1.5	1.65	1.73	1.64	1.49	1.55	1.06
***EARL***_**(0,0.7]**_	95.60	87.60	83.69	79.26	73.08	**64.24**
***EARL***_**(0.7,1.5]**_	3.01	2.72	2.55	2.21	2.46	**1.39**
***EARL***_**(0,1.5]**_	46.22	42.33	40.42	38.17	35.41	**30.72**
***L***	3.3890	3.3800	3.1999	2.8699	2.9995	3.1100

**Table 7:** The OOC *ARL* profiles of the proposed HWMA, DHWMA and HHWMA *W* schemes against the CUSUM, EWMA and DEWMA *W* schemes when (*m*,*n*,*λ*) = (100,5,0.5) for a nominal *ARL*_0_ = 500 under *GAM*(3,1) distribution.

Shift (*δ*)	CUSUM *W*	EWMA *W*	DEWMA *W*	HWMA *W*	DHWMA *W*	HHWMA *W*
0.1	462.44	459.92	457.33	421.30	539.19	314.94
0.2	236.67	221.27	215.71	203.76	148.82	109.49
0.3	73.00	69.08	66.50	56.90	21.82	13.12
0.4	28.93	26.63	24.44	26.54	14.25	7.48
0.5	11.91	11.41	11.00	12.58	9.24	4.32
0.6	8.14	7.88	7.73	7.63	6.08	2.80
0.7	5.77	5.52	5.30	5.16	4.95	2.07
0.8	5.19	4.94	4.60	3.80	3.82	1.99
0.9	4.44	4.11	3.74	3.04	3.39	1.31
1.0	4.00	3.77	3.11	2.56	2.78	1.30
1.1	3.51	3.34	2.60	2.22	2.56	1.20
1.2	3.00	2.79	2.22	1.99	2.21	1.06
1.3	2.72	2.44	2.00	1.81	2.05	1.03
1.4	2.09	2.00	1.77	1.68	1.99	1.01
1.5	1.68	1.58	1.66	1.41	1.70	1.00
***EARL***_**(0,0.7]**_	118.12	114.53	112.57	104.84	106.34	**64.89**
***EARL***_**(0.7,1.5]**_	3.33	3.12	2.71	2.31	2.56	**1.24**
***EARL***_**(0,1.5]**_	56.90	55.11	53.98	50.16	50.99	**30.94**
***L***	3.3890	3.3800	3.1999	2.8699	2.9995	3.1100

Table 5 shows that under the *N*(0,1) distribution, the *EARL*_(0,0.7]_ = 100.89, 96.98, 94.90, 93.31, 86.92 and 80.19 for the CUSUM, EWMA, DEWMA, HWMA, DHWMA and HHWMA *W* schemes, respectively. This reveals that the HHWMA *W* scheme performs better compared to other competing schemes for small shifts. For the moderate shifts, it can be seen that the HHWMA *W* scheme outperforms the competing schemes and it is followed by the HWMA *W* scheme. For instance, the *EARL*_(0.7,1.5]_ are 1.64 and 2.62 for the HHWMA and HWMA *W* schemes, respectively, while the ones for other competing schemes are greater than 2.70. For small-to-moderate shifts, the *EARL*_(0,1.5]_ = 48.54, 46.90, 45.80, 44.94, 42.07 and 38.30 for the CUSUM, EWMA, DEWMA, HWMA, DHWMA and HHWMA *W* schemes, respectively, which reveals that the HHWMA *W* scheme is superior over all competing schemes and it is followed by the DHWMA and HWMA *W* schemes in that order. Table 6 presents the OOC performance comparison of the competing schemes under the *t*(5) distribution. Similar findings to the ones found for the comparison under the *N*(0,1) distribution is also observed under the *t*(5) distribution. In a nutshell, the HHWMA *W* scheme outperforms all competing schemes considered in this paper regardless of the magnitude (or size) of shifts, i.e. small, moderate, large (not shown here to conserve space) and small-to-moderate. For small shifts as well as for small-to-moderate shifts, the HHWMA *W* scheme performs better followed by the DHWMA *W* scheme. For moderate shifts, the HHWMA *W* scheme is superior over the competing schemes followed by the HWMA *W* scheme. Table 7 displays the OOC performance comparison of the competing schemes under the *GAM*(3,1) distribution. From Table 7, it can be observed that the HHWMA *W* scheme outperforms all competing schemes considered in this paper regardless of the magnitude of the shift in the process mean followed by the HWMA *W* scheme.

## 5. Illustrative example

**Fig 7:**
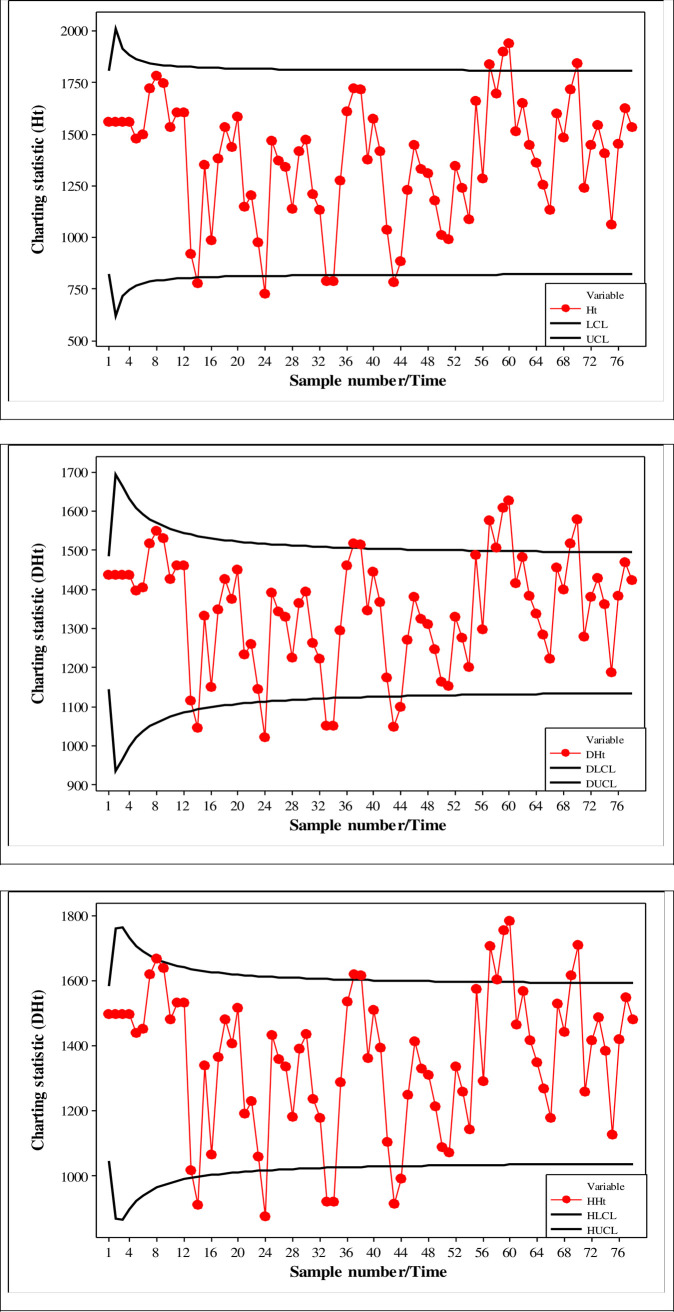
HWMA, DHWMA and HHWMA monitoring schemes of the iron ore date when *m* = 520, *n* = 5, *λ* = 0.5, *λ*_1_ = 0.75 and *λ*_2_ = 0.5 for and nominal *ARL*_0_ value of 500.

In this section, the application of the proposed HWMA, DHWMA and HHWMA *W* monitoring schemes is illustrated using mining data to monitor the percentage of the silicon dioxide in iron ore used by [37]. High concentration of silica is a sign of contamination or impurity and as a result, it is undesirable. Consequently, it is crucial to incessantly monitor the silica concentration in iron ore on the flotation process in order to manage and resolve any peculiarities that may occur. On that note, this paper uses the proposed monitoring schemes to control the level of the percentage of silica in iron ore. The iron ore mining data used in this section consists of two subsets considered as phase I and phase II samples. In this example, it is assumed that a sample of size 5 is drawn every hour. The chi-square test of normality show that the iron ore data is not normally distributed at 5% level of significance (*p-value* = 0.00). Thus, nonparametric schemes are good choice to monitor these data. The phase I data consist of 104 samples of size 5 (*m* = 520) collected when the process was assumed to be IC. In phase II, 78 samples of size 5 each (i.e., *n* = 5) are monitored. The proposed HWMA and DHWMA *W* schemes are implemented using *λ* = 0.5 and it was found that *L*_*H*_ = 2.9069 and *L*_*DH*_ = 2.0095, respectively, for a nominal *ARL*_0_ = 500. The HHWMA *W* scheme is implemented using *λ*_1_ = 0.75 and *λ*_2_ = 0.5 and it was found that *L*_*HH*_ = 2.1171 so that the attained *ARL*_0_ is much closer to the nominal *ARL*_0_ = 500. The plots of the proposed HWMA, DHWMA and HHWMA *W* monitoring schemes are shown in Fig 7A–7C. Table 8 presents the charting statistics and control limits as well as the signalling and non-signalling events of the proposed monitoring schemes when (*m*,*n*) = (520,5) for a nominal *ARL*_0_ = 500. The control limits of the proposed HWMA, DHWMA and HHWMA *W* schemes are denoted as (*LCL*_*t*_, *LCL*_*t*_), (*DLCL*_*t*_, *DLCL*_*t*_) and (*HLCL*_*t*_, *HLCL*_*t*_), respectively, and the OOC signals are boldfaced in Table 8. The findings in both Fig 7 and Table 8 show that the HHWMA *W* scheme gives a signal for the first time on the 8^th^ subgroup in the prospective phase, while the DHWMA and HWMA *W* schemes give a signal for the first time on the 14^th^ subgroup in the prospective phase. This example confirms the superiority of the proposed HHWMA *W* scheme over the HWMA and DHWMA *W* schemes (see Fig 7 and Table 8).

**Table 8:** Illustration example of the proposed HWMA, DHWMA and HHWMA *W* charts using the mining iron ores data with (*m*,*n*,*λ*,*λ*_1_,*λ*_2_) = (520, 5, 0.5, 0.75, 0.5) for a nominal *ARL*_0_ = 500.

Sample number	HWMA *W* scheme				DHWMA *W* scheme				HHWMA *W* scheme			
	*H*_*t*_	*LCL*_*t*_	*UCL*_*t*_	OOC	*DH*_*t*_	*DLCL*_*t*_	*DUCL*_*t*_	OOC	*HH*_*t*_	*HLCL*_*t*_	*HUCL*_*t*_	OOC
1	1557.50	824.33	1805.67	No	1436.25	1145.40	1484.60	No	1496.88	1046.98	1583.02	No
2	1557.50	621.09	2008.91	No	1436.25	935.77	1694.23	No	1496.88	868.31	1761.69	No
3	1557.50	714.06	1915.94	No	1436.25	965.37	1664.63	No	1496.88	866.08	1763.92	No
4	1557.50	748.42	1881.58	No	1436.25	997.71	1632.29	No	1496.88	898.62	1731.38	No
5	1479.00	766.41	1863.59	No	1397.00	1020.74	1609.26	No	1438.00	922.92	1707.08	No
6	1495.75	777.50	1852.50	No	1405.38	1037.53	1592.47	No	1450.56	940.66	1689.34	No
7	1722.25	785.02	1844.98	No	1518.63	1050.27	1579.73	No	1620.44	954.03	1675.97	No
8	1784.50	790.45	1839.55	No	1549.75	1060.26	1569.74	No	1667.13	964.42	1665.58	**Yes**
9	1745.00	794.57	1835.43	No	1530.00	1068.31	1561.69	No	1637.50	972.72	1657.28	No
10	1534.75	797.79	1832.21	No	1424.88	1074.95	1555.05	No	1479.81	979.51	1650.49	No
11	1605.50	800.38	1829.62	No	1460.25	1080.51	1549.49	No	1532.88	985.15	1644.85	No
12	1605.50	802.51	1827.49	No	1460.25	1085.24	1544.76	No	1532.88	989.92	1640.08	No
13	917.75	804.30	1825.71	No	1116.38	1089.32	1540.68	No	1017.06	994.01	1635.99	No
14	778.25	805.81	1824.19	**Yes**	1046.63	1092.88	1537.12	**Yes**	912.44	997.54	1632.46	**Yes**
15	1349.00	807.11	1822.89	No	1332.00	1096.00	1534.00	No	1340.50	1000.64	1629.36	No
16	983.50	808.24	1821.76	No	1149.25	1098.77	1531.23	No	1066.38	1003.37	1626.63	No
17	1380.75	809.23	1820.77	No	1347.88	1101.24	1528.76	No	1364.31	1005.79	1624.21	No
18	1535.00	810.11	1819.89	No	1425.00	1103.46	1526.54	No	1480.00	1007.96	1622.04	No
19	1436.75	810.89	1819.11	No	1375.88	1105.47	1524.53	No	1406.31	1009.91	1620.09	No
20	1583.75	811.58	1818.42	No	1449.38	1107.29	1522.71	No	1516.56	1011.67	1618.33	No
21	1149.00	812.21	1817.79	No	1232.00	1108.95	1521.05	No	1190.50	1013.28	1616.72	No
22	1202.50	812.78	1817.22	No	1258.75	1110.47	1519.53	No	1230.63	1014.74	1615.26	No
23	974.75	813.30	1816.70	No	1144.88	1111.86	1518.14	No	1059.81	1016.08	1613.92	No
24	728.50	813.78	1816.22	**Yes**	1021.75	1113.15	1516.85	**Yes**	875.13	1017.32	1612.68	**Yes**
25	1470.00	814.21	1815.79	No	1392.50	1114.35	1515.65	No	1431.25	1018.46	1611.54	No
26	1371.50	814.61	1815.39	No	1343.25	1115.45	1514.55	No	1357.38	1019.51	1610.49	No
27	1342.75	814.98	1815.02	No	1328.88	1116.49	1513.51	No	1335.81	1020.49	1609.51	No
28	1138.00	815.33	1814.67	No	1226.50	1117.45	1512.55	No	1182.25	1021.40	1608.60	No
29	1416.00	815.65	1814.35	No	1365.50	1118.35	1511.65	No	1390.75	1022.26	1607.75	No
30	1473.75	815.94	1814.06	No	1394.38	1119.19	1510.81	No	1434.06	1023.05	1606.95	No
31	1207.75	816.22	1813.78	No	1261.38	1119.98	1510.02	No	1234.56	1023.80	1606.20	No
32	1131.00	816.48	1813.52	No	1223.00	1120.73	1509.27	No	1177.00	1024.50	1605.50	No
33	788.75	816.72	1813.28	**Yes**	1051.88	1121.43	1508.57	**Yes**	920.31	1025.16	1604.84	**Yes**
34	788.75	816.95	1813.05	**Yes**	1051.88	1122.09	1507.91	**Yes**	920.31	1025.78	1604.22	**Yes**
35	1277.00	817.17	1812.83	No	1296.00	1122.72	1507.28	No	1286.50	1026.37	1603.63	No
36	1607.75	817.37	1812.63	No	1461.38	1123.31	1506.69	No	1534.56	1026.93	1603.07	No
37	1721.50	817.56	1812.44	No	1518.25	1123.88	1506.12	**Yes**	1619.88	1027.45	1602.55	**Yes**
38	1716.75	817.74	1812.26	No	1515.88	1124.41	1505.59	**Yes**	1616.31	1027.95	1602.05	**Yes**
39	1377.50	817.92	1812.08	No	1346.25	1124.92	1505.08	No	1361.88	1028.43	1601.57	No
40	1574.75	818.08	1811.92	No	1444.88	1125.41	1504.59	No	1509.81	1028.88	1601.12	No
41	1418.25	818.24	1811.77	No	1366.63	1125.87	1504.13	No	1392.44	1029.31	1600.69	No
42	1035.75	818.38	1811.62	No	1175.38	1126.31	1503.69	No	1105.56	1029.72	1600.28	No
43	780.25	818.52	1811.48	**Yes**	1047.63	1126.74	1503.26	**Yes**	913.94	1030.11	1599.89	**Yes**
44	883.75	818.66	1811.34	No	1099.38	1127.14	1502.86	**Yes**	991.56	1030.49	1599.51	**Yes**
45	1226.75	818.79	1811.21	No	1270.88	1127.53	1502.47	No	1248.81	1030.84	1599.16	No
46	1446.75	818.91	1811.09	No	1380.88	1127.90	1502.10	No	1413.81	1031.19	1598.81	No
47	1332.75	819.03	1810.97	No	1323.88	1128.25	1501.75	No	1328.31	1031.52	1598.48	No
48	1307.75	819.14	1810.86	No	1311.38	1128.59	1501.41	No	1309.56	1031.83	1598.17	No
49	1179.75	819.25	1810.75	No	1247.38	1128.92	1501.08	No	1213.56	1032.13	1597.87	No
50	1011.00	819.35	1810.65	No	1163.00	1129.24	1500.76	No	1087.00	1032.42	1597.58	No
51	991.25	819.45	1810.55	No	1153.13	1129.54	1500.46	No	1072.19	1032.70	1597.30	No
52	1344.75	819.54	1810.46	No	1329.88	1129.83	1500.17	No	1337.31	1032.97	1597.03	No
53	1239.00	819.64	1810.37	No	1277.00	1130.12	1499.88	No	1258.00	1033.23	1596.77	No
54	1086.75	819.72	1810.28	No	1200.88	1130.39	1499.61	No	1143.81	1033.48	1596.52	No
55	1661.25	819.81	1810.19	No	1488.13	1130.65	1499.35	No	1574.69	1033.72	1596.28	No
56	1282.50	819.89	1810.11	No	1298.75	1130.90	1499.10	No	1290.63	1033.95	1596.05	No
57	1838.00	819.97	1810.03	**Yes**	1576.50	1131.15	1498.85	**Yes**	1707.25	1034.18	1595.82	**Yes**
58	1697.25	820.05	1809.95	No	1506.13	1131.38	1498.62	**Yes**	1601.69	1034.39	1595.61	**Yes**
59	1900.50	820.12	1809.88	**Yes**	1607.75	1131.61	1498.39	**Yes**	1754.13	1034.60	1595.40	**Yes**
60	1938.75	820.19	1809.81	**Yes**	1626.88	1131.83	1498.17	**Yes**	1782.81	1034.81	1595.19	**Yes**
61	1513.25	820.26	1809.74	No	1414.13	1132.05	1497.95	No	1463.69	1035.00	1595.00	No
62	1650.75	820.33	1809.68	No	1482.88	1132.26	1497.74	No	1566.81	1035.19	1594.81	No
63	1449.00	820.39	1809.61	No	1382.00	1132.46	1497.54	No	1415.50	1035.38	1594.62	No
64	1360.50	820.45	1809.55	No	1337.75	1132.65	1497.35	No	1349.13	1035.56	1594.44	No
65	1253.75	820.51	1809.49	No	1284.38	1132.84	1497.16	No	1269.06	1035.73	1594.27	No
66	1132.25	820.57	1809.43	No	1223.63	1133.03	1496.98	No	1177.94	1035.90	1594.10	No
67	1598.25	820.63	1809.37	No	1456.63	1133.20	1496.80	No	1527.44	1036.06	1593.94	No
68	1484.75	820.68	1809.32	No	1399.88	1133.38	1496.62	No	1442.31	1036.22	1593.78	No
69	1717.75	820.74	1809.26	No	1516.38	1133.55	1496.45	**Yes**	1617.06	1036.37	1593.63	**Yes**
70	1841.25	820.79	1809.21	**Yes**	1578.13	1133.71	1496.29	**Yes**	1709.69	1036.52	1593.48	**Yes**
71	1241.00	820.84	1809.16	No	1278.00	1133.87	1496.13	No	1259.50	1036.66	1593.34	No
72	1448.00	820.89	1809.11	No	1381.50	1134.02	1495.98	No	1414.75	1036.81	1593.19	No
73	1543.00	820.93	1809.07	No	1429.00	1134.18	1495.82	No	1486.00	1036.94	1593.06	No
74	1408.00	820.98	1809.02	No	1361.50	1134.32	1495.68	No	1384.75	1037.08	1592.92	No
75	1062.50	821.03	1808.97	No	1188.75	1134.47	1495.53	No	1125.63	1037.21	1592.79	No
76	1454.25	821.07	1808.93	No	1384.63	1134.61	1495.40	No	1419.44	1037.33	1592.67	No
77	1624.50	821.11	1808.89	No	1469.75	1134.74	1495.26	No	1547.13	1037.46	1592.54	No
78	1534.50	821.15	1808.85	No	1424.75	1134.87	1495.13	No	1479.63	1037.58	1592.42	No

## 6. Conclusion

This paper introduced the HWMA, DHWMA and HHWMA monitoring schemes based on the Wilcoxon rank sum *W* statistic. The DHWMA *W* (HHWMA *W*) scheme is the extension of the HWMA *W* scheme where the same (different) smoothing parameter(s) are applied twice. The performances of the proposed schemes in terms of the *RL* characteristics were evaluated using the Monte Carlo simulations in SAS®9.4/IML15.4 with 20000 simulations. It was found that the proposed schemes are IC robust and present very interesting properties regardless of the nature of the underlying probability distribution. The HHWMA *W* scheme is preferred over the HWMA and DHWMA *W* schemes because of its flexibility and sensitivity in monitoring shifts of different sizes. In terms of the *EARL* and *ESDRL* profile, regardless of the value of smoothing parameters, the larger the phase I or/and sample size, the better the overall performance. Therefore, a balance must be strike in order to choose rational sample sizes to avoid high implementation and inspection costs. Compared to the existing CUSUM, EWMA and DEWMA *W* schemes in zero-state, it is observed that the proposed HHWMA scheme outperforms the existing schemes considered in this paper regardless of the magnitude of the shift in the process mean parameter.

From the results of this study, we recommend that quality engineers should put the proposed monitoring schemes into use when monitoring the location parameter regardless of the nature of the probability distribution. For future research, researchers who are interested in the design of efficient and robust schemes can look at the design of the HWMA, DHWMA and HHWMA *W* schemes with fast initial response features. Also, other complex extended HWMA monitoring schemes based on nonparametric statistics can be studied, such as those in [42]. Researchers are also advised to study more advanced schemes based on multi-objective cluster head selection using fitness averaged rider optimization algorithm for IoT networks in smart cities, see for instance [52]. Finally, given some concerns raised in [53], a steady-state mode performances of the proposed schemes need to be examined.

## Supporting information

S1 Appendix Properties of the HWMA *W* scheme. (DOCX)

S2 Appendix Properties of the DHWMA *W* scheme. (DOCX)

S3 Appendix Properties of the HHWMA *W* chart. (DOCX)
