# Supplementary material for: New extended distribution-free homogenously weighted monitoring schemes for monitoring abrupt shifts in the location parameter
Source: PLoS One. 2022 Jan 21;17(1):e0261217. doi: 10.1371/journal.pone.0261217 (PMC8782475; doi:10.1371/journal.pone.0261217)
Supplement: S1 Appendix — (DOCX) [file pone.0261217.s001.docx]

**S1 Appendix: Properties of the HWMA *W* scheme**

This appendix contains the derivations of the mean and variance of the $H_{t}$ statistic. The charting statistic of $H_{t}$ is given by

| $H_{t}=\lambda W_{t}+\left( 1-\lambda\right)\bar{W}_{t-1}$ |  |
| --- | --- |
| where | (A.1) |
| $\bar{W}_{t-1}=\frac{\sum_{k}^{t-1} W_{k}}{t-1}.$ |  |

- For $t=1$,

$$H_{1}=\lambda W_{1}+\left( 1-\lambda\right)\bar{W}_{0}.$$

Since $\bar{W}_{0}=\mu_{W}$, when $t=$ 1, Equation (A.1) becomes:

| $H_{1}=\lambda W_{1}+\left( 1-\lambda\right)\mu_{W}.$ | (A.2) |
| --- | --- |

Thus, the mean and variance of $H_{1}$ are given by

| ${E(H}_{1})=\lambda{E(W}_{1})+\left( 1-\lambda\right)\mu_{W}=\left( \lambda+1-\lambda\right)\mu_{W}=\mu_{W}$ |  |
| --- | --- |
| and | (A.3) |
| ${Var(H}_{1})=Var\left( {\lambda W}_{1}+\left( 1-\lambda\right)\mu_{W} \right)=\lambda^{2}Var\left( W_{1} \right)=\lambda^{2}\sigma_{W}^{2},$ |  |

respectively.

- For $t>1$,

Equation (A.1) can be written as:

| $H_{t}=\lambda W_{t}+\left( 1-\lambda\right)\frac{\sum_{k}^{t-1} W_{k}}{t-1}.$ | (A.4) |
| --- | --- |

From Equation (A.4), when $t>$ 1, the mean of $H_{t}$ can be derived as follows:

$${E(H}_{t})=\lambda{E(W}_{t})+\left( 1-\lambda\right)\frac{\sum_{k}^{t-1} {E(W}_{k})}{t-1}$$

$$=\lambda\mu_{W}+\left( 1-\lambda\right)\frac{E\left( W_{1} \right)+E\left( W_{2} \right)+\ldots+E\left( W_{t-1} \right)}{t-1}$$

$$=\lambda\mu_{W}+\left( 1-\lambda\right)\frac{{\left( t-1 \right)\mu}_{W}}{t-1}$$

$$=\lambda\mu_{W}+\left( 1-\lambda\right) \mu_{W}$$

$$=\mu_{W}.$$

Next, from Equation (A.4), when $t>$ 1, the variance of $H_{t}$ is derived as follows:

$${Var(H}_{t})=Var\left[ {\lambda W}_{t}+\left( 1-\lambda\right)\frac{\sum_{k}^{t-1} W_{k}}{t-1} \right]$$

$$={\lambda^{2}Var(W}_{t})+\left( 1-\lambda\right)^{2}\frac{\sum_{k}^{t-1} {Var (W}_{k})}{t-1}$$

$$=\lambda^{2}\sigma_{W}^{2}+\left( \frac{1-\lambda}{t-1} \right)^{2}\left( {Var (W}_{1})+{Var (W}_{2})+\ldots+{Var (W}_{t-1}) \right)$$

$$=\lambda^{2}\sigma_{W}^{2}+\left( \frac{1-\lambda}{t-1} \right)^{2}\left( t-1 \right)\sigma_{W}^{2}$$

$$=\left( \lambda^{2}+\frac{\left( 1-\lambda\right)^{2}}{t-1} \right)\sigma_{W}^{2}.$$

Hence, the mean and variance of the $H_{t}$ statistic are given by

| $E(H_{t})=\mu_{H_{t}}=\mu_{W}$ | (A.5) |
| --- | --- |
| and |  |
| $Var\left( H_{t} \right)=\sigma_{H_{t}}^{2}=\left\{ \begin{aligned} \lambda^{2}\sigma_{W}^{2} for t=1 \\ \left[ \lambda^{2}+\frac{{(1-\lambda)}^{2}}{t-1} \right]\sigma_{W}^{2}, for t>1, \end{aligned} \right.$ |  |

respectively.
