# Supplementary material for: New extended distribution-free homogenously weighted monitoring schemes for monitoring abrupt shifts in the location parameter
Source: PLoS One. 2022 Jan 21;17(1):e0261217. doi: 10.1371/journal.pone.0261217 (PMC8782475; doi:10.1371/journal.pone.0261217)
Supplement: S2 Appendix — (DOCX) [file pone.0261217.s002.docx]

**S2 Appendix: Properties of the DHWMA *W* scheme**

This Appendix contains the derivations of the mean and variance of the ${DH}_{t}$ statistic. The charting statistic of $DH_{t}$ is given by

| $\left\{ \begin{aligned} H_{t}=\lambda W_{t}+(1-\lambda)\bar{W}_{t-1} \\ {DH}_{t}=\lambda H_{t}+(1-\lambda)\bar{H}_{t-1} \end{aligned} \right.$ |  |
| --- | --- |
| with | (B.1) |
| $\bar{W}_{t-1}=\frac{\sum_{k}^{t-1} W_{k}}{t-1}$ and $\bar{H}_{t-1}=\frac{\sum_{k}^{t-1} H_{k}}{t-1}.$ |  |

- For $t=1$,

| ${DH}_{1}=\lambda H_{1}+\left( 1-\lambda\right)\bar{H}_{0}=\lambda^{2}W_{1}+\lambda\left( 1-\lambda\right)\bar{W}_{0}+\left( 1-\lambda\right)\bar{H}_{0}.$ | (B.2) |
| --- | --- |

Since $\bar{W}_{0}=\bar{H}_{0}=\mu_{W}$, when $t=$ 1, Equation (B.2) becomes:

| ${DH}_{1}=\lambda^{2}W_{1}+\left[ \lambda\left( 1-\lambda\right)+1-\lambda\right]\mu_{W}=\lambda^{2}W_{1}+\left( 1-\lambda^{2} \right)\mu_{W}.$ | (B.3) |
| --- | --- |

Thus, the mean and variance of ${DH}_{1}$ are given by

| ${E(DH}_{1})=\lambda^{2}{E(W}_{1})+\left( 1-\lambda^{2} \right)\mu_{W}=\lambda^{2}\mu_{W}+\left( 1-\lambda^{2} \right)\mu_{W}=\mu_{W}$ |  |
| --- | --- |
| and | (B.4) |
| ${Var(DH}_{1})=Var\left[ \lambda^{2}W_{1}+\left( 1-\lambda^{2} \right)\mu_{W} \right]=\lambda^{4}Var\left( W_{1} \right)=\lambda^{4}\sigma_{W}^{2},$ |  |

respectively.

- For $t=2$,

$${DH}_{2}=\lambda H_{2}+\left( 1-\lambda\right)\bar{H}_{1}$$

$$=\lambda\left( \lambda W_{2}+\left( 1-\lambda\right)\bar{W}_{1} \right)+\left( 1-\lambda\right)\bar{H}_{1}$$

$$=\lambda^{2}W_{2}+\lambda\left( 1-\lambda\right)W_{1}+\left( 1-\lambda\right)\left[ \lambda W_{1}+\left( 1-\lambda\right)\mu_{W} \right]$$

$$=\lambda^{2}W_{2}+2\lambda\left( 1-\lambda\right)W_{1}+\left( 1-\lambda\right)^{2}\mu_{W}.$$

Thus, the expression of ${DH}_{2}$ is given by

| ${DH}_{2}=\lambda^{2}W_{2}+2\lambda\left( 1-\lambda\right)W_{1}+\left( 1-\lambda\right)^{2}\mu_{W}.$ | (B.5) |
| --- | --- |

From Equation (B.5), when $t=$ 2, the mean of ${DH}_{t}$ can be derived as follows:

$${E(DH}_{2})=\lambda^{2}{E(W}_{2})+2\lambda\left( 1-\lambda\right)E(W_{1})+\left( 1-\lambda\right)^{2}\mu_{W}$$

$$=\left[ \lambda^{2}+2\lambda\left( 1-\lambda\right)+\left( 1-\lambda\right)^{2} \right]\mu_{W}$$

$$=\left[ \lambda^{2}+2\lambda-2\lambda^{2}+1-2\lambda+\lambda^{2} \right]\mu_{W}$$

$$=\mu_{W}.$$

From Equation (B.5), when $t=$ 2, the variance of ${DH}_{t}$ can be derived as follows:

$${Var(DH}_{2})=Var[\lambda^{2}W_{2}+2\lambda\left( 1-\lambda\right)W_{1}+\left( 1-\lambda\right)^{2}\mu_{W}]$$

$$=\lambda^{4}{Var(W}_{2})+4\lambda^{2}\left( 1-\lambda\right)^{2}Var(W_{1})$$

$$=\lambda^{2}\left[ \lambda^{2}+4\left( 1-\lambda\right)^{2} \right]\sigma_{W}^{2}.$$

Thus, the mean and variance of ${DH}_{2}$ are given by

| ${E(DH}_{2})=\mu_{W}$ |  |
| --- | --- |
| and | (B.6) |
| ${Var(DH}_{2})=\lambda^{2}\left[ \lambda^{2}+4\left( 1-\lambda\right)^{2} \right]\sigma_{W}^{2},$ |  |

respectively.

- For $t>2$,

$${DH}_{t}=\lambda H_{t}+\left( 1-\lambda\right)\bar{H}_{t-1}$$

$$=\lambda\left. \left( \lambda W_{t}+\left( 1-\lambda\right)\bar{W}_{t-1} \right. \right)+\frac{\left( 1-\lambda\right)}{t-1}\sum_{k=1}^{t-1} H_{k}$$

$$=\lambda^{2}W_{t}+\lambda\left( 1-\lambda\right)\bar{W}_{t-1}+\frac{\left( 1-\lambda\right)}{t-1}\sum_{k=1}^{t-1} (\lambda W_{k}+(1-\lambda)\bar{W}_{k-1})$$

$$=\lambda^{2}W_{t}+\lambda\left( 1-\lambda\right)\bar{W}_{t-1}+\frac{\lambda\left( 1-\lambda\right)}{t-1}\sum_{k=1}^{t-1} W_{k}+\frac{\left( 1-\lambda\right)^{2}}{t-1}\sum_{k=1}^{t-1} \bar{W}_{k-1}$$

$$=\lambda^{2}W_{t}+2\lambda\left( 1-\lambda\right)\bar{W}_{t-1}+\frac{({1-\lambda)}^{2}}{t-1}\sum_{k=0}^{t-2} \bar{W}_{k}$$

$$=\lambda^{2}W_{t}+2\lambda\left( 1-\lambda\right)\bar{W}_{t-1}+\frac{({1-\lambda)}^{2}}{t-1}\sum_{k=1}^{t-2} \bar{W}_{k}+\frac{({1-\lambda)}^{2}}{t-1}\mu_{W}$$

$$=\lambda^{2}W_{t}+2\lambda\left( 1-\lambda\right)\bar{W}_{t-1}+\frac{({1-\lambda)}^{2}}{t-1}\sum_{k=1}^{t-2} \frac{1}{k}\sum_{u=1}^{t-2} W_{u}+\frac{({1-\lambda)}^{2}}{t-1}\mu_{W}$$

$$=\lambda^{2}W_{t}+\frac{2\lambda\left( 1-\lambda\right)}{t-1}W_{t-1}+\frac{2\lambda\left( 1-\lambda\right)}{t-1}\sum_{u=1}^{t-2} W_{u}+\frac{({1-\lambda)}^{2}}{t-1}\sum_{u=1}^{t-2} \left( \sum_{k=u}^{t-2} \left. \frac{1}{k} \right)W_{u} \right.+\frac{({1-\lambda)}^{2}}{t-1}\mu_{W}$$

$$=\lambda^{2}W_{t}+\frac{2\lambda\left( 1-\lambda\right)}{t-1}W_{t-1}+\frac{\left( 1-\lambda\right)}{t-1}\sum_{u=1}^{t-2} \left( 2\lambda+\left( 1-\lambda\right)\sum_{k=u}^{t-2} \left. \frac{1}{k} \right) \right.W_{u}+\frac{({1-\lambda)}^{2}}{t-1}\mu_{W}.$$

Thus, the expression of ${DH}_{t}$ is given by

| ${DH}_{t}=\lambda^{2}W_{t}+\frac{2\lambda\left( 1-\lambda\right)}{t-1}W_{t-1}+\frac{\left( 1-\lambda\right)}{t-1}\sum_{u=1}^{t-2} \left( 2\lambda+\left( 1-\lambda\right)\sum_{k=u}^{t-2} \left. \frac{1}{k} \right) \right.W_{u}+\frac{({1-\lambda)}^{2}}{t-1}\mu_{W}.$ | (B.7) |
| --- | --- |

From Equation (B.7), when $t>$ 2, the mean of ${DH}_{t}$ can be derived as follows:

$$E\left( {DH}_{t} \right)=\left[ \lambda^{2}+\frac{2\lambda\left( 1-\lambda\right)}{t-1}+\frac{\left( 1-\lambda\right)}{t-1}\sum_{u=1}^{t-2} \left( 2\lambda+\left( 1-\lambda\right)\sum_{k=u}^{t-2} \left. \frac{1}{k} \right) \right.+\frac{({1-\lambda)}^{2}}{t-1} \right]\mu_{W}$$

$$=(\lambda^{2}+\frac{2\lambda\left( 1-\lambda\right)}{t-1}+\frac{2\lambda\left( 1-\lambda\right)\left( t-2 \right)}{t-1}+({1-\lambda)}^{2})\mu_{W}$$

$$=(\lambda^{2}+2\lambda\left( 1-\lambda\right)+({1-\lambda)}^{2})\mu_{W}$$

$$=\mu_{W}.$$

From Equation (B.7), when $t>$ 2, the variance of ${DH}_{t}$ can be derived as follows:

$${Var(DH}_{t})=Var\left[ \lambda^{2}W_{t}+\frac{2\lambda\left( 1-\lambda\right)}{t-1}W_{t-1}+\frac{\left( 1-\lambda\right)}{t-1}\sum_{u=1}^{t-2} \left( 2\lambda+\left( 1-\lambda\right)\sum_{k=u}^{t-2} \left. \frac{1}{k} \right) \right.W_{u}+\frac{({1-\lambda)}^{2}}{t-1}\mu_{W} \right]$$

$$=\lambda^{4}Var(W_{t})+\frac{4\lambda^{2}\left( 1-\lambda\right)^{2}}{\left( t-1 \right)^{2}}{Var(W}_{t-1})+\frac{\left( 1-\lambda\right)^{2}}{\left( t-1 \right)^{2}}\sum_{u=1}^{t-2} \left( 2\lambda+\left( 1-\lambda\right)\sum_{k=u}^{t-2} \left. \frac{1}{k} \right) \right.^{2}{Var(W}_{u})$$

$$=\left. \left[ \lambda^{4}+\frac{4\lambda^{2}\left( 1-\lambda\right)^{2}}{\left( t-1 \right)^{2}}+\frac{\left( 1-\lambda\right)^{2}}{\left( t-1 \right)^{2}}\sum_{u=1}^{t-2} \left( 2\lambda+\left( 1-\lambda\right)\sum_{k=u}^{t-2} \left. \frac{1}{k} \right) \right.^{2} \right. \right]\sigma_{W}^{2}.$$

Thus, when $t>$ 2, the mean and variance of ${DH}_{t}$ are given by

| $E({DH}_{t})=\mu_{W}$ | (B.8) |
| --- | --- |
| and |  |
| ${Var(DH}_{t})=\left. \left[ \lambda^{4}+\frac{4\lambda^{2}\left( 1-\lambda\right)^{2}}{{(t-1)}^{2}}+\frac{\left( 1-\lambda\right)^{2}}{{(t-1)}^{2}}\sum_{u=1}^{t-2} \left( 2\lambda+\left( 1-\lambda\right)\sum_{k=u}^{t-2} \left. \frac{1}{k} \right) \right.^{2} \right. \right]\sigma_{W,}^{2}$ |  |

respectively.

Therefore, at the sampling time $t$, the mean and variance of ${DH}_{t}$ statistic are defined by

| $E({DH}_{t})=\mu_{W}$ | (B.9) |
| --- | --- |
| and |  |
| $Var\left( {DH}_{t} \right)=\left\{ \begin{aligned} \lambda^{4}\sigma_{W}^{2} for t=1 \\ \lambda^{2}\left( \lambda^{2}+4\left( 1-\lambda\right)^{2} \right)\sigma_{W}^{2} for t=2 \\ \left[ \lambda^{4}+\frac{4\lambda^{2}\left( 1-\lambda\right)^{2}}{\left( t-1 \right)^{2}} \right.+\frac{\left( 1-\lambda\right)^{2}}{\left( t-1 \right)^{2}}\left. {\sum_{u=1}^{t-2} \left( 2\lambda+\left( 1-\lambda\right)\sum_{k=u}^{t-2} \frac{1}{k} \right)}^{2} \right]\sigma_{W}^{2} for t>2, \end{aligned} \right.$ |  |

respectively.
