# Supplementary material for: New extended distribution-free homogenously weighted monitoring schemes for monitoring abrupt shifts in the location parameter
Source: PLoS One. 2022 Jan 21;17(1):e0261217. doi: 10.1371/journal.pone.0261217 (PMC8782475; doi:10.1371/journal.pone.0261217)
Supplement: S3 Appendix — (DOCX) [file pone.0261217.s003.docx]

**S3 Appendix: Properties of the HHWMA *W* chart**

This Appendix contains the derivations of the mean and variance of the ${HH}_{t}$ statistic. The charting statistic of ${HH}_{t}$ is given by

| $\left\{ \begin{aligned} H_{t}=\lambda_{2}W_{t}+(1-\lambda_{2})\bar{W}_{t-1} \\ {HH}_{t}=\lambda_{1}H_{t}+(1-\lambda_{1})\bar{H}_{t-1} \end{aligned} \right.$ | (C.1) |
| --- | --- |
| where |  |
| $\bar{W}_{t-1}=\frac{\sum_{k}^{t-1} W_{k}}{t-1}$ and $\bar{H}_{t-1}=\frac{\sum_{k}^{t-1} H_{k}}{t-1}.$ |  |

- For$t=1$,

| ${HH}_{1}=\lambda_{1}H_{1}+\left( 1-\lambda_{1} \right)\bar{H}_{0} =\lambda_{1}\left[ \lambda_{2}W_{1}+\left( 1-\lambda_{2} \right)\bar{W}_{0} \right]+\left( 1-\lambda_{1} \right)\bar{H}_{0}.$ | (C.2) |
| --- | --- |

Since $\bar{W}_{0}=\bar{H}_{0}=\mu_{W}$, when $t=$ 1, Equation (C.2) becomes:

| ${HH}_{1}=\lambda_{1}\left[ \lambda_{2}W_{1}+\left( 1-\lambda_{2} \right)\mu_{W} \right]+\left( 1-\lambda_{1} \right)\mu_{W}=\lambda_{1}\lambda_{2}W_{1}+({1-\lambda}_{1}\lambda_{2})\mu_{W}.$ | (C.3) |
| --- | --- |

Thus, the mean and variance of ${HH}_{1}$ are given by

| $E({HH}_{1})=\lambda_{1}\lambda_{2}{E(W}_{1})+({1-\lambda}_{1}\lambda_{2})\mu_{W}{=\lambda}_{1}\lambda_{2}\mu_{W}+\mu_{W}-\lambda_{1}\lambda_{2}\mu_{W}=\mu_{W}$ |  |
| --- | --- |
| and | (C.4) |
| $Var({HH}_{1})=Var(\lambda_{1}\lambda_{2}W_{1}+\left( {1-\lambda}_{1}\lambda_{2} \right)\mu_{W})=\lambda_{1}^{2}\lambda_{2}^{2}{Var(W}_{1})=\lambda_{1}^{2}\lambda_{2}^{2}\sigma_{W}^{2},$ |  |

respectively.

For $t=2$,

$${HH}_{2}=\lambda_{1}H_{2}+\left( 1-\lambda_{1} \right)\bar{H}_{1}$$

$$=\lambda_{1}\left[ \lambda_{2}W_{2}+\left( 1-\lambda_{2} \right)\bar{W}_{1} \right]+\left( 1-\lambda_{1} \right)\bar{H}_{1}$$

$$=\lambda_{1}\lambda_{2}W_{2}+\lambda_{1}\left( 1-\lambda_{2} \right)W_{1}+\left( 1-\lambda_{1} \right)\left[ \lambda_{2}W_{1}+\left( 1-\lambda_{2} \right)\bar{W}_{0} \right]$$

$$=\lambda_{1}\lambda_{2}W_{2}+\lambda_{1}\left( 1-\lambda_{2} \right)W_{1}+\lambda_{2}\left( 1-\lambda_{1} \right)W_{1}+\left( 1-\lambda_{1} \right)\left( 1-\lambda_{2} \right)\mu_{W}$$

$$=\lambda_{1}\lambda_{2}W_{2}+(\lambda_{1}+\lambda_{2}-2\lambda_{1}\lambda_{2})W_{1}+\left( 1-\lambda_{1} \right)\left( 1-\lambda_{2} \right)\mu_{W.}$$

Thus, the expression of ${HH}_{2}$ is given by

| ${HH}_{2}=\lambda_{1}\lambda_{2}W_{2}+(\lambda_{1}+\lambda_{2}-2\lambda_{1}\lambda_{2})W_{1}+\left( 1-\lambda_{1} \right)\left( 1-\lambda_{2} \right)\mu_{W} .$ | (C.5) |
| --- | --- |

From Equation (C.5), when $t=$ 2, the mean of ${HH}_{t}$ can be derived as follows:

$$E({HH}_{2})=\lambda_{1}\lambda_{2}{E(W}_{1})+(\lambda_{1}+\lambda_{2}-2\lambda_{1}\lambda_{2}){E(W}_{1})+\left( 1-\lambda_{1} \right)\left( 1-\lambda_{2} \right)\mu_{W}$$

$$=\left[ \lambda_{1}\lambda_{2}+\lambda_{1}+\lambda_{2}-2\lambda_{1}\lambda_{2}+1-\lambda_{2}-\lambda_{1}+\lambda_{1}\lambda_{2} \right]\mu_{W}$$

$$=\mu_{W}.$$

From Equation (C.5), when $t=$ 2, the variance of ${HH}_{t}$ can be derived as follows:

$Var\left( {HH}_{2} \right)=\lambda_{1}^{2}\lambda_{2}^{2}Var\left( W_{2} \right)+{{(\lambda}_{1}+\lambda_{2}-2\lambda_{1}\lambda_{2})}^{2}Var\left( W_{1} \right)=\left[ \lambda_{1}^{2}\lambda_{2}^{2}+{{(\lambda}_{1}+\lambda_{2}-2\lambda_{1}\lambda_{2})}^{2} \right]\sigma_{W}^{2}.$

Thus, the mean and variance of ${DH}_{2}$ are given by

| ${E(HH}_{2})=\mu_{W}$ |  |
| --- | --- |
| and | (C.6) |
| ${Var(HH}_{2})=[\lambda_{1}^{2}\lambda_{2}^{2}+{{(\lambda}_{1}+\lambda_{2}-2\lambda_{1}\lambda_{2})}^{2}]\sigma_{W}^{2},$ |  |

respectively.

- For $t>2$,

$${HH}_{t}=\lambda_{1}H_{t}+\left( 1-\lambda_{1} \right)\bar{H}_{t-1}$$

$$=\lambda_{1}\left[ \lambda_{2}W_{t}+\left( 1-\lambda_{2} \right)\bar{W}_{t-1} \right]+\frac{\left( 1-\lambda_{1} \right)}{t-1}\sum_{k=1}^{t-1} H_{t}$$

$$=\lambda_{1}\lambda_{2}W_{t}+\lambda_{1}(1-\lambda_{2})\bar{W}_{t-1}+\frac{\left( 1-\lambda_{1} \right)}{t-1}\sum_{k=1}^{t-1} {[\lambda}_{2}W_{k}+(1-\lambda_{2})\bar{W}_{k-1}]$$

$$=\lambda_{1}\lambda_{2}W_{t}+\lambda_{1}\left( 1-\lambda_{2} \right)\bar{W}_{t-1}+\frac{\lambda_{2}\left( 1-\lambda_{1} \right)}{t-1}\sum_{k=1}^{t-1} W_{k}+\frac{\left( 1-\lambda_{1} \right)\left( 1-\lambda_{2} \right)}{t-1}\sum_{k=1}^{t-1} \bar{W}_{k-1}$$

$$=\lambda_{1}\lambda_{2}W_{t}+\left( \lambda_{1}+\lambda_{2}-2\lambda_{1}\lambda_{2} \right)W_{t-1}+\frac{\left( 1-\lambda_{1} \right)\left( 1-\lambda_{2} \right)}{t-1}\sum_{k=0}^{t-2} \bar{W}_{k}$$

$$=\lambda_{1}\lambda_{2}W_{t}+\left( \lambda_{1}+\lambda_{2}-2\lambda_{1}\lambda_{2} \right)\bar{W}_{t-1}+\frac{\left( 1-\lambda_{1} \right)\left( 1-\lambda_{2} \right)}{t-1}\sum_{k=1}^{t-2} \bar{W}_{k}+\frac{\left( 1-\lambda_{1} \right)\left( 1-\lambda_{2} \right)}{t-1}\mu_{W}$$

$$=\lambda_{1}\lambda_{2}W_{t}+\left( \lambda_{1}+\lambda_{2}-2\lambda_{1}\lambda_{2} \right)\bar{W}_{t-1}+\frac{\left( 1-\lambda_{1} \right)\left( 1-\lambda_{2} \right)}{t-1}\sum_{u=1}^{t-2} \frac{1}{k}\sum_{k=u}^{t-2} W_{u}+\frac{\left( 1-\lambda_{1} \right)\left( 1-\lambda_{2} \right)}{t-1}\mu_{W}$$

$$=\lambda_{1}\lambda_{2}W_{t}+\left( \lambda_{1}+\lambda_{2}-2\lambda_{1}\lambda_{2} \right)\bar{W}_{t-1}+\frac{\left( 1-\lambda_{1} \right)\left( 1-\lambda_{2} \right)}{t-1}\sum_{u=1}^{t-2} \left. \left( \sum_{k=u}^{t-2} \frac{1}{k} \right. \right)W_{u}+\frac{\left( 1-\lambda_{1} \right)\left( 1-\lambda_{2} \right)}{t-1}\mu_{W}$$

$$=\lambda_{1}\lambda_{2}W_{t}+\frac{\left( \lambda_{1}+\lambda_{2}-2\lambda_{1}\lambda_{2} \right)}{t-1}W_{t-1}+\frac{\left( \lambda_{1}+\lambda_{2}-2\lambda_{1}\lambda_{2} \right)}{t-1}\sum_{u=1}^{t-2} W_{u}+\frac{\left( 1-\lambda_{1} \right)\left( 1-\lambda_{2} \right)}{t-1}\sum_{u=1}^{t-2} \left. \left( \sum_{k=u}^{t-2} \frac{1}{k} \right. \right)W_{u}+\frac{\left( 1-\lambda_{1} \right)\left( 1-\lambda_{2} \right)}{t-1}\mu_{W}$$

$$=\lambda_{1}\lambda_{2}W_{t}+\frac{\left( \lambda_{1}+\lambda_{2}-2\lambda_{1}\lambda_{2} \right)}{t-1}W_{t-1}+\frac{1}{t-1}\sum_{u=1}^{t-2} \left[ \left. \left( \lambda_{1}+\lambda_{2}-2\lambda_{1}\lambda_{2} \right)+\left( 1-\lambda_{1} \right)(1-\lambda_{2})\sum_{k=u}^{t-2} \frac{1}{k} \right] \right.W_{u}+\frac{\left( 1-\lambda_{1} \right)(1-\lambda_{2})}{t-1}\mu_{W}.$$

Thus, the expression of ${HH}_{t}$ is given by

| ${HH}_{t}=\lambda_{1}\lambda_{2}W_{t}+\frac{\left( \lambda_{1}+\lambda_{2}-2\lambda_{1}\lambda_{2} \right)}{t-1}W_{t-1}+\frac{1}{t-1}\sum_{u=1}^{t-2} \left[ \left. \left( \lambda_{1}+\lambda_{2}-2\lambda_{1}\lambda_{2} \right)+\left( 1-\lambda_{1} \right)(1-\lambda_{2})\sum_{k=u}^{t-2} \frac{1}{k} \right] \right.W_{u}+\frac{\left( 1-\lambda_{1} \right)(1-\lambda_{2})}{t-1}\mu_{W}.$ | (C.7) |
| --- | --- |

From Equation (C.7), when $t>$ 2, the mean of ${HH}_{t}$ can be derived as follows:

$$E\left( {HH}_{t} \right)=\left[ \lambda_{1}\lambda_{2}+\frac{\left( \lambda_{1}+\lambda_{2}-2\lambda_{1}\lambda_{2} \right)}{t-1}+\frac{1}{t-1}\sum_{u=1}^{t-2} \left[ \left. \left( \lambda_{1}+\lambda_{2}-2\lambda_{1}\lambda_{2} \right)+\left( 1-\lambda_{1} \right)\left( 1-\lambda_{2} \right)\sum_{k=u}^{t-2} \frac{1}{k} \right] \right.+\frac{\left( 1-\lambda_{1} \right)\left( 1-\lambda_{2} \right)}{t-1} \right]\mu_{W}$$

$$=\left. \left[ \lambda_{1}\lambda_{2}+\frac{\left( \lambda_{1}+\lambda_{2}-2\lambda_{1}\lambda_{2} \right)}{t-1}+\frac{\left( \lambda_{1}+\lambda_{2}-2\lambda_{1}\lambda_{2} \right)\left( t-2 \right)}{t-1}+\frac{\left( 1-\lambda_{1} \right)\left( 1-\lambda_{2} \right)}{t-1}\sum_{u=1}^{t-2} \left[ \left. +\sum_{k=u}^{t-2} \frac{1}{k} \right] \right.+\frac{\left( 1-\lambda_{1} \right)\left( 1-\lambda_{2} \right)}{t-1} \right. \right]\mu_{W}=\left[ \left. \lambda_{1}\lambda_{2}+\lambda_{1}+\lambda_{2}-2\lambda_{1}\lambda_{2}+\left( 1-\lambda_{1} \right)\left( 1-\lambda_{2} \right) \right]\mu_{W} \right.$$

$$=\mu_{W}.$$

From Equation (C.7), when $t>$ 2, the variance of ${HH}_{t}$ can be derived as follows:

$${Var(HH}_{t})=Var\left[ \lambda_{1}\lambda_{2}W_{t}+\frac{\left( \lambda_{1}+\lambda_{2}-2\lambda_{1}\lambda_{2} \right)}{t-1}W_{t-1}+\frac{1}{t-1}\sum_{u=1}^{t-2} \left[ \left. \left( \lambda_{1}+\lambda_{2}-2\lambda_{1}\lambda_{2} \right)+\left( 1-\lambda_{1} \right)\left( 1-\lambda_{2} \right)\sum_{k=u}^{t-2} \frac{1}{k} \right] \right.W_{u}+\frac{\left( 1-\lambda_{1} \right)\left( 1-\lambda_{2} \right)}{t-1}\mu_{W} \right]$$

$$=\left[ \left. \lambda_{1}^{2}\lambda_{2}^{2}+\frac{{{(\lambda}_{1}+\lambda_{2}-2\lambda_{1}\lambda_{2})}^{2}}{\left( t-1 \right)^{2}}+\frac{1}{\left( t-1 \right)^{2}}\sum_{u=1}^{t-2} \left( \lambda_{1}+\lambda_{2}-2\lambda_{1}\lambda_{2}+\left( 1-\lambda_{1} \right)\left( 1-\lambda_{2} \right)\sum_{k=u}^{t-2} \frac{1}{k} \right)^{2} \right] \right.\sigma_{W}^{2}.$$

Thus, when $t>$ 2, the mean and variance of ${HH}_{t}$ are given by

| $E({HH}_{t})=\mu_{W}$ | (C.8) |
| --- | --- |
| and |  |
| ${Var(HH}_{t})=\left. \left[ \lambda_{1}^{2}\lambda_{2}^{2}+\frac{{{(\lambda}_{1}+\lambda_{2}-2\lambda_{1}\lambda_{2})}^{2}}{\left( t-1 \right)^{2}}+\frac{1}{\left( t-1 \right)^{2}}\sum_{u=1}^{t-2} \left( \lambda_{1}+\lambda_{2}-2\lambda_{1}\lambda_{2}+\left( 1-\lambda_{1} \right)\left( 1-\lambda_{2} \right)\sum_{k=u}^{t-2} \frac{1}{k} \right)^{2} \right. \right]\sigma_{W}^{2},$ |  |

respectively.

Therefore, at the sampling time $t$, the mean and variance of ${HH}_{t}$ statistic are defined by

| $E({HH}_{t})=\mu_{W}$ | (C.9) |
| --- | --- |
| and |  |
| $Var\left( {HH}_{t} \right)=\left\{ \begin{aligned} \lambda_{1}^{2}\lambda_{2}^{2}\sigma_{W}^{2} \mathrm{for} t=1 \\ \left( \lambda_{1}^{2}\lambda_{2}^{2}+{{(\lambda}_{1}+\lambda_{2}-2\lambda_{1}\lambda_{2})}^{2} \right)\sigma_{W}^{2} \mathrm{for} t=2 \\ \left[ \lambda_{1}^{2}\lambda_{2}^{2}+\frac{{{(\lambda}_{1}+\lambda_{2}-2\lambda_{1}\lambda_{2})}^{2}}{\left( t-1 \right)^{2}}+\left. \frac{1}{\left( t-1 \right)^{2}}\sum_{u=1}^{t-2} \left( \lambda_{1}+\lambda_{2}-2\lambda_{1}\lambda_{2}+\left( 1-\lambda_{1} \right)\left( 1-\lambda_{2} \right)\sum_{k=u}^{t-2} \frac{1}{k} \right)^{2} \right]\sigma_{W}^{2} \right. \mathrm{for} t>2 \end{aligned} \right.$ |  |

respectively. Finally, note that when $\lambda_{1}=\lambda_{2}$, then the results are the same as that of ${DH}_{t}$ statistic.
